# How fluorine substituents strengthen aryl C–H bonds

**DOI:** 10.1039/d6sc01846g

**Published:** 2026-04-27

**Authors:** Daniel A. Santos Oliveira, Daniela Rodrigues Silva, Ataualpa A. C. Braga, Célia Fonseca Guerra, Robin N. Perutz, Odile Eisenstein, F. Matthias Bickelhaupt

**Affiliations:** a Department of Chemistry and Pharmaceutical Sciences, AIMMS, Vrije Universiteit Amsterdam De Boelelaan 1108 Amsterdam 1081 HZ The Netherlands f.m.bickelhaupt@vu.nl https://www.theochem.nl; b Department of Fundamental Chemistry, Institute of Chemistry, University of São Paulo Av. Prof. Lineu Prestes, 748 São Paulo 055508-000 Brazil; c Department of Chemistry, University of York York YO10 5DD UK robin.perutz@york.ac.uk; d ICGM, Univ. Montpellier, CNRS, ENSCM Montpellier France odile.eisenstein@umontpellier.fr; e Department of Chemistry and Hylleraas Centre for Quantum Molecular Sciences, University of Oslo PO Box 1033 Blindern Oslo 0315 Norway; f Institute of Molecules and Materials, Radboud University Heyendaalseweg 135 Nijmegen 6525 AJ The Netherlands; g Department of Chemical Sciences, University of Johannesburg Auckland Park Johannesburg 2006 South Africa

## Abstract

We have investigated the nature and bond dissociation energies (BDEs) of the aromatic C–H bonds in fluorinated benzenes C_6_R_5_H (each R can be H or F) using quantitative Kohn–Sham molecular orbital theory and a matching energy decomposition analysis (EDA). The C–H bond becomes stronger as the number of fluorine atoms in the benzene ring increases. This increase in the calculated BDE is additive and most pronounced for *ortho*-substituted C–H bonds. Our analyses of the C–H bond between 
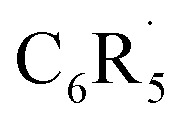
 and H˙ reveal that a fluorine inductive effect is responsible for this. Fluorine polarizes the closed-shell molecular orbitals of 
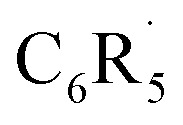
 away from the carbon radical center and in this way reduces Pauli repulsion between [C˙] and the H˙ radical, leading to a stronger C–H bond. The *ortho* effect can be accurately modelled by a combination of Pauli repulsion (main contribution) and orbital interactions. We extend our analysis to other substituents, including ones with the opposite effect on C–H bond strength.

## Abstract 2

A reduction in Pauli repulsion is the major determinant of increased bond dissociation energy on fluorine substitution, with an additional contribution from orbital interactions required to account for the *ortho* selectivity.

## Graphical abstract

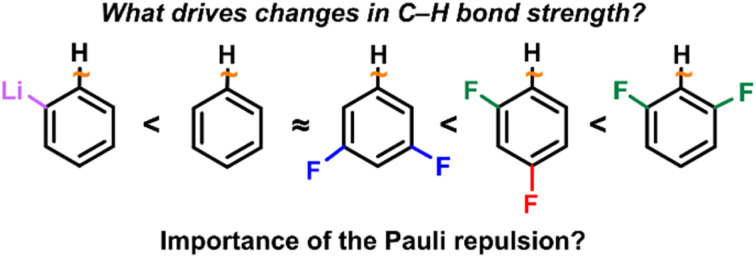

## Introduction

Fluorine alters the physical properties of chemical species. For instance, it modifies the lipophilicity and acid–base properties, leading to the adoption of fluorine-containing organic compounds, including numerous F- or CF_3_-substituted aromatics, in pharmaceuticals, imaging, agrochemicals, and many other areas.^1*a*–*g*^ In materials science, fluorinated conducting materials and fluorinated anions are important, especially in rechargeable batteries.^1*i*–*k*^ Through its deep influence on the molecular dipole and quadrupole, it can serve as a conformational tool in organic and biological chemistry.^1*l*^ The formation of the halogen bond is strongly associated with fluorinated organic molecules such as iodopentafluorobenzene.^1*m*^ Through the modification of intermolecular interactions, it enhances charge mobilities in organic semiconductors.^1*n*^ Zooming in on some fundamental properties of fluorinated benzenes and polyaromatics, fluorine has been found to decrease the aromaticity of aromatic rings while enhancing π-stacking properties. However, the decrease in aromaticity of the ring is accompanied by increased thermostability and resistance to chemical attack, a phenomenon known as fluoroaromaticity.^1*o*–*r*^ The alternating stacking of benzene·hexafluorobenzene cocrystals and related cocrystals in a parallel but displaced geometry was originally attributed to interactions of the different quadrupoles of the two components. Although electrostatics make a major contribution to the interactions, the displaced geometry is suggested to result from Pauli repulsion and dispersion forces, illustrating the effectiveness of Energy Decomposition Analysis in these systems.^1*s*^

Another key effect of fluorine substitution is its impact on the strength of aryl C–H bonds, typically quantified by the homolytic bond dissociation energy (BDE), which can be modulated by both the number and the position of fluorine atoms.^2^ Several groups have investigated the correlations between C–H BDEs in (poly)fluorinated benzenes and C–metal bonds in corresponding metal aryl derivatives, demonstrating that the C–H bond strength increases upon fluorination.^3^ For instance, the calculated C–H bond dissociation energy (BDE) for pentafluorobenzene (C_6_F_5_H: 123.7 kcal mol^−1^) is about 6 kcal mol^−1^ higher than that for benzene (C_6_H_6_: 117.7 kcal mol^−1^). Perutz, Eisenstein, Jones and their co-workers have also found that the most significant increases in bond strength occur when the fluorine atom is in the *ortho* position relative to the C–H bond analyzed.^4^ For those reactions that are thermodynamically driven, particularly reversible reactions, this can result in *ortho* regioselectivity as demonstrated in numerous experimental studies.^3^ These findings are intriguing, since many examples in the literature have shown that neighboring bulky groups tend to weaken the C–H bond through steric repulsion,^5^ but it is unclear how this will apply to fluorine whose van der Waals radius is only slightly larger than that of hydrogen. Although the bond dissociation energies of (poly)fluorinated benzenes have been reported, a detailed explanation, firmly grounded in quantum mechanics, of why fluorine substituents strengthen C–H bonds in fluorinated benzenes is still lacking in the literature.

**Fig. 1:**
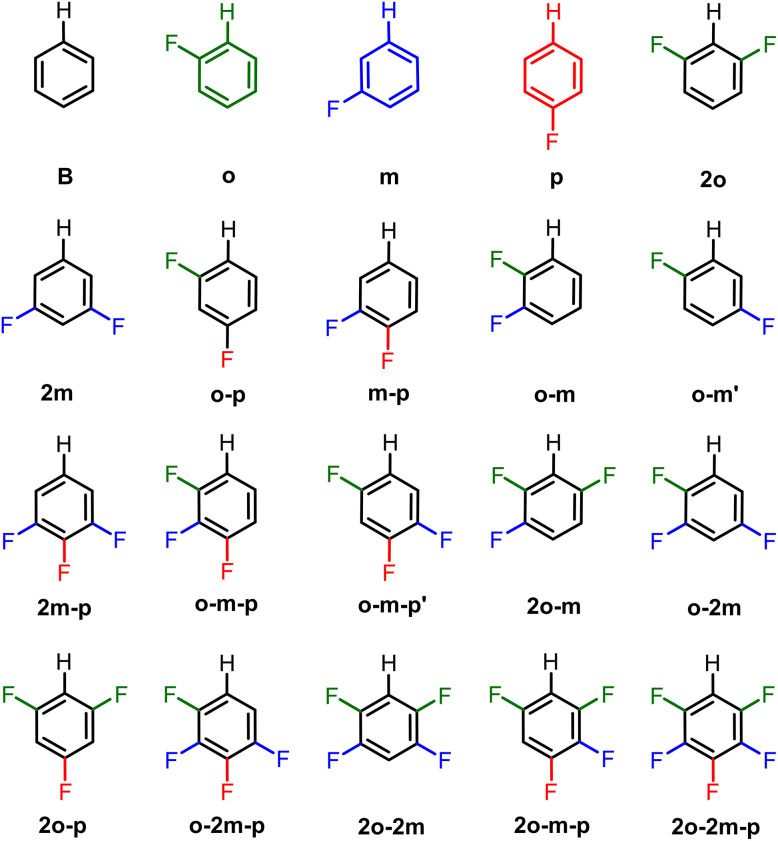
Fluorinated benzene C_6_R_5_H (R = H, F) systems studied in this work.

In this work, we unravel the physical mechanism underlying the strengthening of C–H bonds in (poly)fluorinated benzenes. We also address the origin of the variation in strengthening of the C–H bonds that follows the order *ortho* ≫ *para* > *meta*.^4^ To this end, we investigate the C–H bonding nature in the systems C_6_R_5_–H (each R can be H or F; see Fig. 1) using quantitative Kohn–Sham molecular orbital theory (KS-MO) combined with a matching energy decomposition analysis (EDA).^6,7^ Our results reveal that the main factor responsible for the strengthening of the C–H bonds upon fluorination is the inductive polarization by fluorine of the closed-shell molecular orbital density away from the aromatic ring. This lowers the Pauli repulsion in the bonding region and thus strengthens the C–H bond. The EDA analysis reveals that the regioselectivity of the C–H bond energies, especially the *ortho*/*para* ratio, can be reproduced in part by the Pauli repulsion but an accurate representation requires the sum of Pauli repulsion and orbital terms. We further include C_6_RH_4_–H systems (R = Cl, Br, I, Li) to assess the effect of different substituents beyond fluorine.

## Computational methods

### Computational details

All calculations were carried out using the Amsterdam Density Functional (ADF) program (ADF2019.3 for Potential Energy Surface (PES) scans and ADF2024.1 for all other computations),^8^ which is part of the Amsterdam Modeling Suite (AMS2024.1).^9^ Geometries and energies were obtained with the BLYP level of the generalized gradient approximation (GGA).^10^ Dispersion interactions were accounted for using the DFT-D3(BJ) method developed by Grimme and coworkers,^11^ which includes the damping function proposed by Becke and Johnson.^12^ Scalar relativistic effects were treated using the zeroth-order regular approximation (ZORA).^13^ Molecular orbitals (MOs) were expanded in a large, uncontracted set of Slater-type orbitals (STOs), specifically the TZ2P basis set, which is of triple-ζ quality and includes two sets of polarization functions.^14^ All electrons were treated variationally. Radical fragments were treated using a spin-unrestricted formalism. The numerical accuracy^15^ was set to VERYGOOD. All optimized structures were confirmed as true minima by vibrational frequency analyses, showing no imaginary frequencies.^16^ The bond energies reported in this work correspond to electronic energies and do not include zero-point energy (ZPE) corrections. Multiple regression analyses were conducted using the statsmodels Python library,^17^ while graphical visualization of the results was performed with the Matplotlib library.^18^ The relative bond enthalpies computed at our final level of theory, ZORA-BLYP-D3(BJ)/TZ2P, are in good agreement with experimentally validated literature values,^19^ supporting the reliability of our methodological approach (see Table S1).

### Activation strain model and energy decomposition analysis

**Scheme 1:**
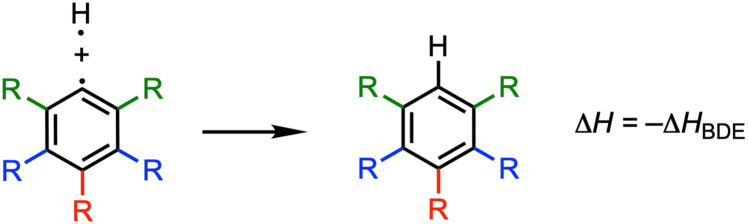
Formation of the C–H bond in C_6_R_5_H (R = H, F).

The overall homolytic C–H bond enthalpy Δ*H*, corresponding to −Δ*H*_BDE_, between 
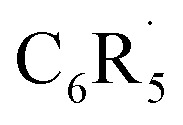
 and H˙ in C_6_R_5_–H (R = H, F), is shown in Scheme 1. The corresponding value of Δ*E* is decomposed into two major components using the activation strain model (ASM, eqn (1)):^20^

1 Δ*E* = Δ*E*_strain_ + Δ*E*_int_

here, the strain energy Δ*E*_strain_ is the energy penalty required to deform the aryl fragment from its equilibrium structure to the geometry that it acquires in the final molecule. The interaction energy Δ*E*_int_ accounts for all chemical interactions between the geometrically deformed fragments in C_6_R_5_–H.

The interaction energy Δ*E*_int_ is further analyzed within the framework of the quantitative Kohn–Sham molecular orbital (KS-MO)^6^ theory by partitioning it using our canonical energy decomposition analysis (EDA) scheme into electrostatic interactions, Pauli repulsion, (attractive) orbital interactions, dispersion corrections, and spin polarization (eqn (2)):^7^

2 Δ*E*_int_ = Δ*V*_elstat_ + Δ*E*_Pauli_ + Δ*E*_oi_ + Δ*E*_disp_ + Δ*E*_spinpol_

The electrostatic energy Δ*V*_elstat_ corresponds to the electrostatic interactions between the unperturbed charge distribution of the distorted fragments, which is usually attractive. The Pauli repulsion Δ*E*_Pauli_ comprises the destabilizing interactions between occupied orbitals (or, more precisely, same-spin electrons on either fragment) and is responsible for any steric repulsion. The orbital interactions Δ*E*_oi_ term, accounts for stabilizing orbital interactions between the fragments including both polarization and overlap effects and can be further decomposed into the electron pair-bond energy Δ*E*_pb_ and Δ*E*_rel_ (eqn (3)). Δ*E*_pb_ is defined as the energy change associated with the formation of a doubly occupied bonding combination of the two SOMOs while all other virtual orbitals are deleted. The relaxation energy Δ*E*_rel_, results from full relaxation after including all virtual orbitals. The Δ*E*_rel_ term includes both charge transfer interactions (donor–acceptor interaction between an occupied orbital of one fragment with an empty orbital of the other fragment) and polarization effects (empty/occupied) orbital mixing on one fragment due to the presence of another fragment.^7^

3 Δ*E*_oi_ = Δ*E*_pb_ + Δ*E*_rel_

The dispersion energy Δ*E*_disp_ is added as a correction.^11^ Finally, the Δ*E*_spinpol_ term refers to the spin polarization of the spin-α and spin-β electrons of the deformed unrestricted fragments and is destabilizing (*i.e.*, the deformed unrestricted fragments without spin polarization lie consistently 2–4 kcal mol^−1^ higher in energy and therefore have a too stabilizing Δ*E*_int_).^21^ The open-source PyFrag2019 program was used to automate analyzing the bonding mechanism as a function of the C–H distance.^22^

## Results and discussion

### General trends in bond strength

The bond enthalpies Δ*H* (see Scheme 1) under standard conditions (298.15 K and 1 atm) and the C–H bond lengths (*r*_C–H_) of the C_6_R_5_–H model systems (R = H, F), obtained from our ZORA-BLYP-D3(BJ)/TZ2P computations, are collected in Table 1. The computed C–H bond strengths as represented by the homolytic bond enthalpies Δ*H*, are in excellent agreement with the previously reported results by Clot, Perutz, Eisenstein, and co-workers,^3*c*,4^ confirming that the introduction of fluorine atoms into the benzene ring strengthens the C–H bond for all substitution patterns. The calculated bond enthalpies relative to benzene (B) vary from ΔΔ*H* = −0.4 kcal mol^−1^ for the *meta*-C–H bond in fluorobenzene (m) to ΔΔ*H* = −6.4 kcal mol^−1^ for 1,3,5-trifluorobenzene (2o-p). Fluorination of the benzene ring also leads to a shortening of the C–H bonds. The variations are very small but are approximately proportional to the bond strengthening, with stronger bonds generally associated with shorter bond lengths (Fig. S1). The largest contraction of Δ*r*_C–H_ = −0.004 Å occurs in 2o-p, *i.e.*, the system with the strongest C–H bond.

**Table 1:** Bond enthalpies and energies (Δ*H* and Δ*E*; in kcal mol^−1^), activation strain model terms (in kcal mol^−1^), and bond lengths (in Å) of the C–H bond in C_6_R_5_H (R = H, F)^a^

System	*r*_C–H_	Δ*H*	ΔΔ*H*	Δ*E*	ΔΔ*E*	Δ*E*_strain_	ΔΔ*E*_strain_	Δ*E*_int_	ΔΔ*E*_int_
B	1.088	−109.3	0.0	−115.9	0.0	1.8	0.0	−117.7	0.0
o	1.086	−111.8	−2.5	−118.4	−2.5	1.8	0.0	−120.2	−2.5
m	1.087	−109.7	−0.4	−116.3	−0.4	1.8	0.0	−118.1	−0.4
p	1.087	−110.6	−1.3	−117.2	−1.3	1.7	−0.1	−118.9	−1.2
o-m	1.087	−111.8	−2.5	−118.3	−2.4	1.9	0.1	−120.2	−2.5
m-p	1.087	−110.7	−1.4	−117.3	−1.4	1.8	0.0	−119.1	−1.4
2o	1.085	−114.8	−5.5	−121.1	−5.2	1.8	0.0	−122.9	−5.2
2m	1.087	−110.2	−0.9	−116.8	−0.9	1.9	0.1	−118.7	−1.0
o-p	1.086	−113.0	−3.7	−119.5	−3.6	1.7	−0.1	−121.2	−3.5
o-m′	1.086	−112.2	−2.9	−118.7	−2.8	1.8	0.0	−120.5	−2.8
o-m-p	1.086	−112.7	−3.4	−119.2	−3.3	1.9	0.1	−121.1	−3.4
2m-p	1.086	−110.9	−1.6	−117.5	−1.6	1.9	0.1	−119.4	−1.7
2o-m	1.085	−114.7	−5.4	−121.1	−5.2	1.8	0.0	−122.9	−5.2
o-2m	1.086	−112.4	−3.1	−118.9	−3.0	1.9	0.1	−120.8	−3.1
o-m-p′	1.085	−113.1	−3.8	−119.6	−3.7	1.8	0.0	−121.4	−3.7
2o-p	1.084	−115.7	−6.4	−122.1	−6.2	1.7	−0.1	−123.8	−6.1
o-2m-p	1.086	−113.0	−3.7	−119.5	−3.6	1.9	0.1	−121.4	−3.7
2o-2m	1.086	−115.0	−5.7	−121.3	−5.4	1.9	0.1	−123.2	−5.5
2o-m-p	1.085	−115.5	−6.2	−121.8	−5.9	1.8	0.0	−123.6	−5.9
2o-2m-p	1.085	−115.5	−6.2	−121.8	−5.9	1.9	0.1	−123.7	−6.0

a Computed at ZORA-BLYP-D3(BJ)/TZ2P, enthalpies Δ*H* at 298.15 K and 1 atm. Energies relative to benzene (B) are given as ΔΔ.

**Fig. 2:**
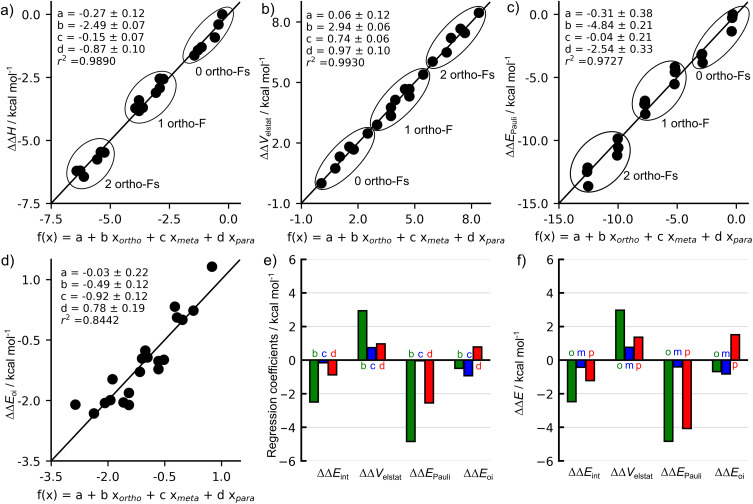
Multiple linear regression relating the number of fluorine substituents at the *ortho*, *meta*, and *para* positions to relative (a) C–H bond enthalpies ΔΔ*H*, (b) electrostatic interactions ΔΔ*V*_elstat_, (c) Pauli repulsions ΔΔ*E*_Pauli_, and (d) orbital interactions ΔΔ*E*_oi_; (e) energy decomposition analysis for all EDA terms from regression coefficients for all 20 fluorinated benzenes relative to benzene; and (f) energy decomposition analysis terms in fluorobenzene relative to benzene at a consistent geometry with a C–H distance of 1.088 Å.^24^ Computed at ZORA-BLYP-D3(BJ)/TZ2P.

As previously reported,^4,23^ and further confirmed here *via* multiple regression analysis, the ΔΔ*H* values can be expressed as a linear function (*r*^2^ = 0.9890) of the number of *ortho*- (*x*_*ortho*_), *meta*- (*x*_*meta*_), and *para*-fluorine substituents (*x*_*para*_), as shown in eqn (4) and Fig. 2a.

4 ΔΔ*H* = *a* + *bx*_*ortho*_ + *cx*_*met*a_ + *dx*_*para*_

The regression coefficients *b*, *c*, and *d*, represent the energy variation associated with the addition of a fluorine atom at the *ortho*, *meta*, and *para* positions, respectively. Accordingly, fluorination at the *ortho* position strengthens the C–H bond by approximately 2.5 ± 0.1 kcal mol^−1^, while *meta*- and *para*-fluorine substitution increase the bond strength by only 0.2 ± 0.1 and 0.9 ± 0.1 kcal mol^−1^, respectively. It is worth noting how closely the regression coefficients calculated here match those reported in the literature^3*c*^ using the B3PW91 functional (*a* = −0.27 ± 0.12 *vs.* −0.17 ± 0.07, *b* = −2.49 ± 0.07 *vs.* −2.49 ± 0.05, *c* = −0.15 ± 0.07 *vs.* −0.07 ± 0.05, and *d* = −0.81 ± 0.07 *vs.* −0.87 ± 0.10 kcal mol^−1^), indicating the robustness of the results with respect to the level of theory. Since these values match so closely with two different functionals, there is no reason to expect significant differences in the EDA analysis. We therefore carried out further analysis with the ZORA-BLYP-D3(BJ)/TZ2P level of theory only.

As shown previously, the ΔΔ*H* values cluster into three groups corresponding to a progressive increase in C–H bond strength as the number of *ortho*-F substituents increases (0 → 1 → 2). Because the *para*-coefficient (*d* = −0.87 kcal mol^−1^) is significant, a secondary subdivision is observed within each region, distinguishing systems that contain a *para*-fluorine from those without a *para*-fluorine. Overall, the linear correlation demonstrates that substituting a fluorine atom for hydrogen at a specific position has an additive effect on the C–H bond strength, with this effect being most pronounced at the *ortho* position, followed by the *para* and *meta* positions, respectively. Here we only analyze the changes in the C–H bond dissociation energy. In contrast,^23^ the C–F bond dissociation energy also changes but in the opposite direction, such that C–F BDEs decrease markedly with the number of *ortho* fluorine substituents, with smaller effects from *meta*- and *para*-fluorine substitution.

The overall trends in bond enthalpies Δ*H* are well reproduced by the electronic bond energies Δ*E*, as shown by the relative ΔΔ values in Table 1 and the multiple regression analyses shown in Fig. S2. Therefore, to elucidate the origin of the C–H bond strengthening in fluorinated benzenes, we analyzed the electronic bond energy Δ*E* using the Activation Strain Model (ASM).^20^ Within this framework, Δ*E* is decomposed into a strain energy Δ*E*_strain_ and an interaction energy Δ*E*_int_ (eqn (1); see Computational methods). The corresponding ASM terms are also listed in Table 1. The only significant geometrical deformation observed upon bond formation is the in-plane bending of the neighboring substituents (H or F) in the aryl fragment. This deformation leads to a small strain energy Δ*E*_strain_, which remains essentially constant across different substitution patterns. Thus, the trends in bond strength (*i.e.*, in both Δ*H* and Δ*E*) are entirely governed by the interaction energy Δ*E*_int_. We further investigated the underlying C–H bonding mechanism using Kohn–Sham Molecular Orbital theory (KS-MO)^6^ and a matching Energy Decomposition Analysis (EDA).^7^ This method decomposes Δ*E*_int_ into distinct physically meaningful components: electrostatic interactions Δ*V*_elstat_, Pauli repulsion Δ*E*_Pauli_, orbital interactions Δ*E*_oi_, among others (see eqn (2) in the Computational methods section). The EDA terms for the C–H bonds in fluorinated benzenes, relative to benzene, can also be expressed as linear functions of the number of *ortho*- (*x*_*ortho*_), *meta*- (*x*_*meta*_), and *para*-fluorine substituents (*x*_*para*_), as depicted in Fig. 2b–d. The corresponding absolute values are provided in Table S1. The graphs in Fig. 2a–c exhibit the characteristic patterns corresponding to the dominance of the *ortho* contribution (*x*_*ortho*_ = 0, 1, or 2) as shown by the rings around the points. Within each subset, we have all possible combinations of *meta*- and *para*-Fs (6 species for 0 and 2 *ortho*-Fs, 7 species for 1 *ortho*-F). The linear correlations indicate that each *para* substitution has a similar effect (see above). The contribution from *meta* substituents is negligible in Fig. 2a and c (ΔΔ*H* and ΔΔ*E*_Pauli_) but is comparable to the *para* contribution for ΔΔ*V*_elstat_. The spans of the electrostatic, Pauli, and orbital contributions are 8.5, 13.6, and 3.6 kcal mol^−1^, respectively (Fig. 2b–d).

**Fig. 3:**
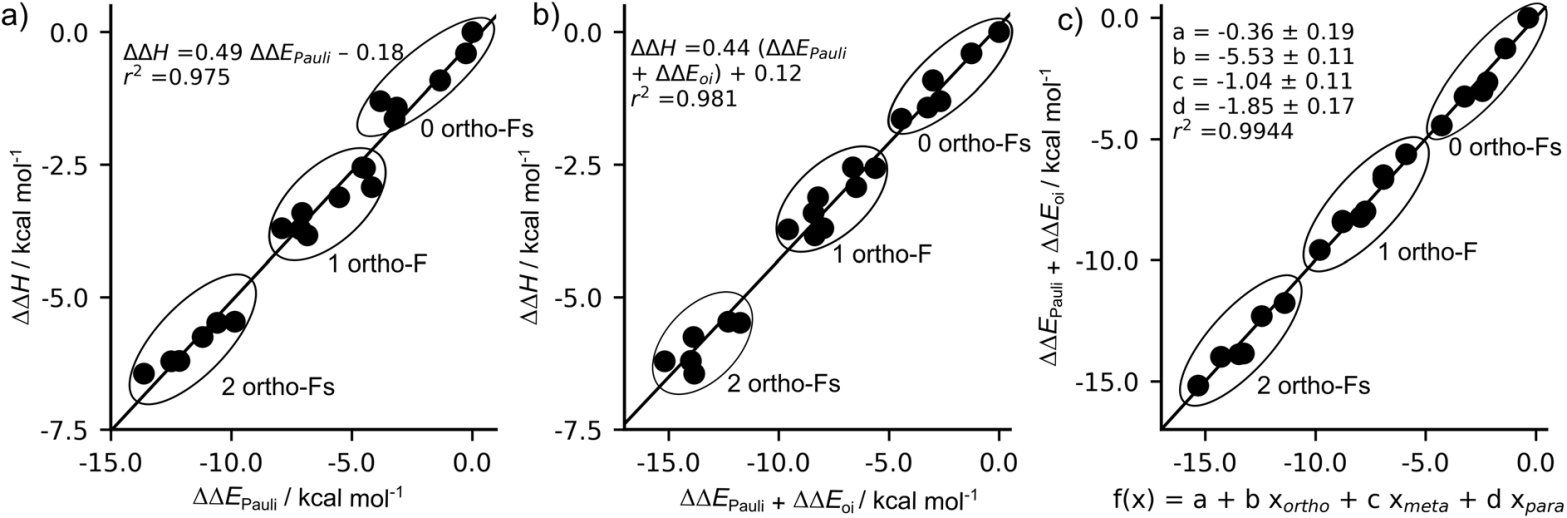
Linear correlation between ΔΔ*H* and (a) ΔΔ*E*_Pauli_, (b) ΔΔ*E*_Pauli_ + ΔΔ*E*_oi_; (c) multiple linear regression relating the number of fluorine substituents at the *ortho*, *meta*, and *para* positions to ΔΔ*E*_Pauli_ + ΔΔ*E*_oi_. Computed at ZORA-BLYP-D3(BJ)/TZ2P.

The trends in the interaction energy, and therefore in the C–H bond enthalpies, are primarily dictated by the Pauli repulsion Δ*E*_Pauli_, as shown in Fig. 2c and e and through linear correlation between ΔΔ*H* and ΔΔ*E*_Pauli_ (Fig. 3a). Relative to benzene, Δ*E*_Pauli_ is the most stabilizing contribution, becoming less repulsive as the number of *ortho*-F substituents increases and exhibiting the same clustering pattern observed for the bond enthalpies (Fig. 2c). Fluorination at the *ortho* position decreases the Pauli repulsion by approximately 4.8 ± 0.2 kcal mol^−1^, while *para*-F substitution reduces Δ*E*_Pauli_ by 2.5 ± 0.3 kcal mol^−1^. The stronger effect of *ortho* compared to *para* substitution is quantified by the ratio *b*/*d*. The value of *b*/*d* for ΔΔ*H* is 2.86 ± 0.33. The corresponding value for ΔΔ*E*_Pauli_ is 1.91 ± 0.26 thus accounting for approximately 2/3 of the *ortho* preference.^25^ Similar to the bond enthalpy, fluorination at the *meta* position has almost no effect on the Pauli repulsion.

In contrast to Δ*E*_Pauli_, the electrostatic interaction term Δ*V*_elstat_ follows the opposite trend to Δ*H*, becoming less attractive as the number of *ortho*-F substituents increases, as indicated by the positive regression coefficients (Fig. 2b). The magnitude of the electrostatic effects follows the pattern *ortho* ≫ *meta* > *para*, which may be ascribed to simple distance effects, 1/*r*, arising because of the 9 electrons of fluorine compared to 1 of hydrogen. Finally, unlike the other EDA terms, the orbital interaction term Δ*E*_oi_ does not exhibit the same clustering pattern based on the number of *ortho*-F substituents and therefore follows a different trend from the bond strength (Fig. 2d). In fact, the stabilization upon fluorine substitution is larger for the *meta* position than for the *ortho* position (−0.9 ± 0.1 *versus* −0.5 ± 0.1 kcal mol^−1^, respectively). In contrast, *para*-F substitution leads to a destabilization of 0.8 ± 0.2 kcal mol^−1^ in the orbital interaction term. This destabilizing contribution at the *para* position accounts for the smaller, remaining difference between the *ortho*- and *para*-C–H bond strengths, as evidenced by the improved correlation coefficient and the closer match between the *b*/*d* ratios obtained from the multiple regressions of ΔΔ*E*_oi_ + ΔΔ*E*_Pauli_ and of ΔΔ*H* (2.99 ± 0.28 and 2.86 ± 0.33, respectively, Fig. 3b and c).

A systematic analysis of the *ortho*-, *meta*- and *para*-fluorine substituent effect on the EDA terms, using different systems as initial references, reveals trends similar to those obtained from the regression coefficients when all 20 C–H bonds are considered (Fig. 2e, f and S5).^26^ Additionally, the same conclusions obtained from the analysis at equilibrium and consistent geometries can be drawn from the analysis of the EDA terms as a function of the C–H bond distance (see Fig. S7 and S8).

Our results discussed so far demonstrate that the Pauli repulsion provides a unified explanation for both the overall C–H bond strengthening in (poly)fluorobenzenes and the major part of the selective increase in bond strength for the C–H bond *ortho* to fluorine, which is fully captured by the combined Pauli and orbital interaction terms. In this way, we address two important open questions of which the underlying causes had remained unknown until now.^2*b*^ A comprehensive analysis of the origin of the Pauli repulsion trends is provided in the following section.

### Origin of the Pauli repulsion

As discussed above, our quantitative MO and EDA analyses reveal that Pauli repulsion Δ*E*_Pauli_ plays a key role in the strengthening of the C–H bonds upon fluorine substitution on the benzene ring. As will become clear in the following, the inductive effect of fluorine polarizes the closed-shell molecular orbitals of the aryl fragment towards the fluorine atom, which reduces their amplitude in the C–H bonding region of the 
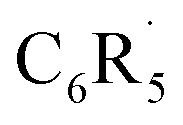
 radical fragment. This, in turn, leads to a decrease in Pauli repulsion with the singly-occupied hydrogen 1s orbital, resulting in a stronger C–H bond.

**Fig. 4:**
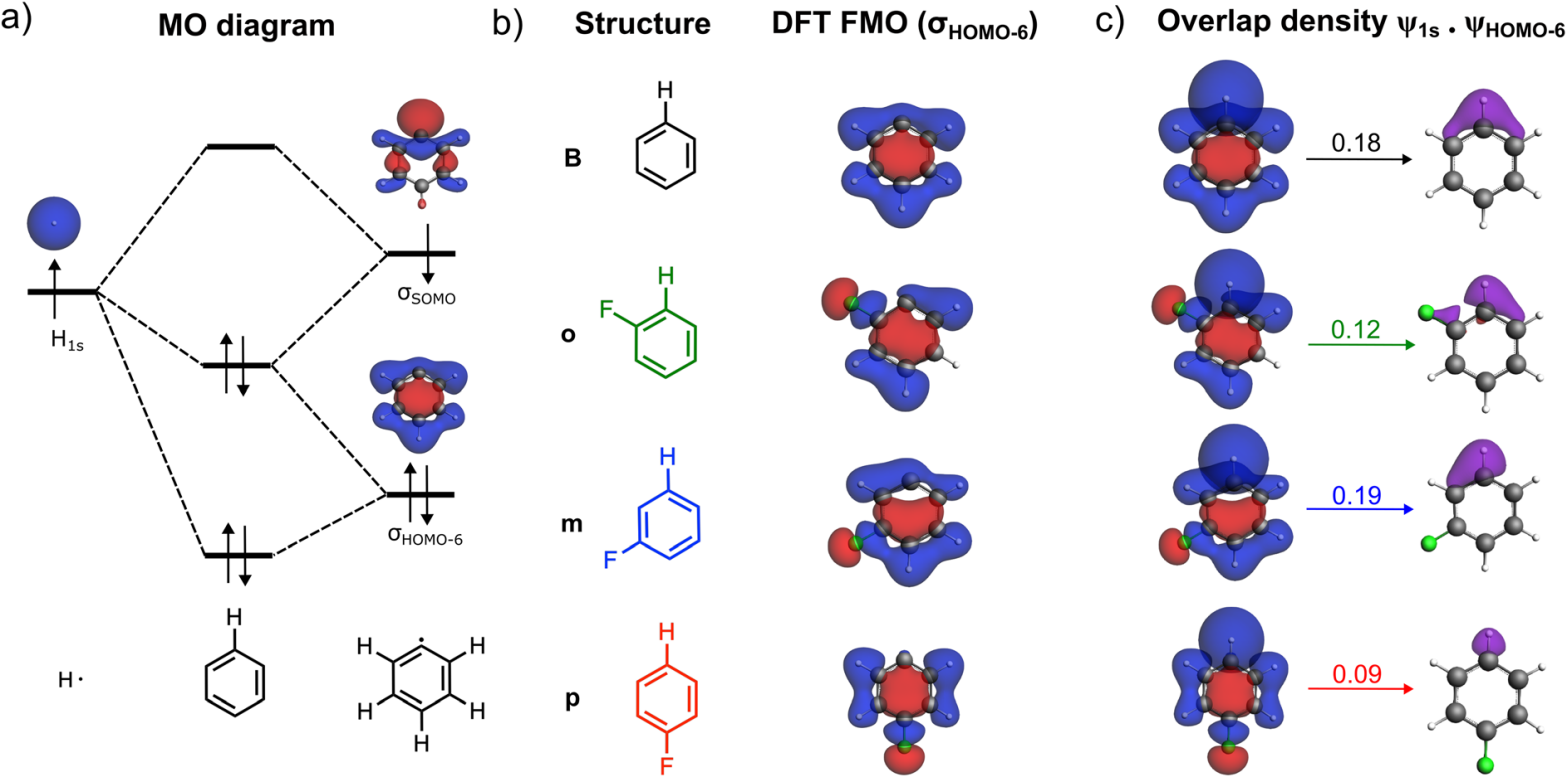
(a) Schematic MO diagram of the C–H bond formation in benzene, (b) *σ*_HOMO−6_ occupied FMO *ψ*_HOMO−6_ (isovalue = 0.03 au) of the aryl fragment, and (c) overlapping FMOs, 〈H_1s_|*σ*_HOMO−6_〉 orbital overlap (above arrows), and overlap density given by the product of fragment orbitals *ψ*_1s_·*ψ*_HOMO−6_ (isovalue = 0.002 au). Computed at ZORA-BLYP-D3(BJ)/TZ2P.

In the EDA framework, the Δ*E*_Pauli_ term arises from repulsive interactions between same-spin occupied orbitals on the respective fragments.^7^ Therefore, all Δ*E*_Pauli_ originates from occupied–occupied orbital interactions between the singly occupied hydrogen 1s orbital and the same-spin component of the occupied molecular orbitals in the σ-electron system of the aryl fragment (see Fig. 4a). The greater the orbital overlap between the hydrogen 1s and the aryl σ-MOs, the stronger the Pauli repulsion. Table S3 lists key overlaps between the hydrogen 1s AO and the σ-MOs of the aryl fragment. The occupied orbital most affected by the presence of a fluorine substituent is the *σ*_HOMO−6_ of the aryl radical (Fig. 4b). The orbital overlap between the hydrogen 1s orbital and the aryl *σ*_HOMO−6_ orbital amounts to 〈H_1s_|*σ*_HOMO−6_〉 = 0.18 for benzene (Fig. 4c). This orbital overlap significantly decreases for the *ortho*- and *para*-C–H bonds, namely to 〈H_1s_|*σ*_HOMO−6_〉 = 0.12 and 0.09, respectively. This leads to the lower Pauli repulsion for the latter two bonds. Note that for the *meta*-C–H bond, the overlap remains nearly unchanged: 〈H_1s_|*σ*_HOMO−6_〉 = 0.19; hence the Δ*E*_Pauli_ value remains similar to that in benzene.

A similar, though less pronounced, effect is observed in the 〈H_1s_|*σ*_HOMO−2_〉 orbital overlap. Fig. S9 presents the 〈H_1s_|*σ*_HOMO−6_〉 and 〈H_1s_|*σ*_HOMO−2_〉 overlaps as a function of the C–H bond distance. Interestingly, although the largest reduction in Pauli repulsion occurs for the *ortho*-C–H bond, the largest decrease in 〈H_1s_|*σ*_HOMO−6_〉 overlap occurs at the *para* position. However, when considering the combined effect of both the 〈H_1s_|*σ*_HOMO−6_〉 and 〈H_1s_|*σ*_HOMO−2_〉, a more significant reduction is observed for the *ortho*-C–H bond, whereas the *para* position shows only a modest reduction in the second overlap. An approximate way to assess the overall contribution of all orbitals to the Pauli repulsion is by considering the sum of the squared overlaps (*S*^2^) between the H_1s_ orbital and all same-spin occupied σ-orbitals of the aryl fragment.^27^ Accordingly, the ∑*S*^2^ values for the C–H bond in benzene and for the *ortho*-, *meta*-, and *para*-C–H bonds in fluorobenzene are 0.244, 0.227, 0.242, and 0.234, respectively. These values follow the same trend observed for both the Pauli repulsion and the C–H bond strength.

**Fig. 5:**
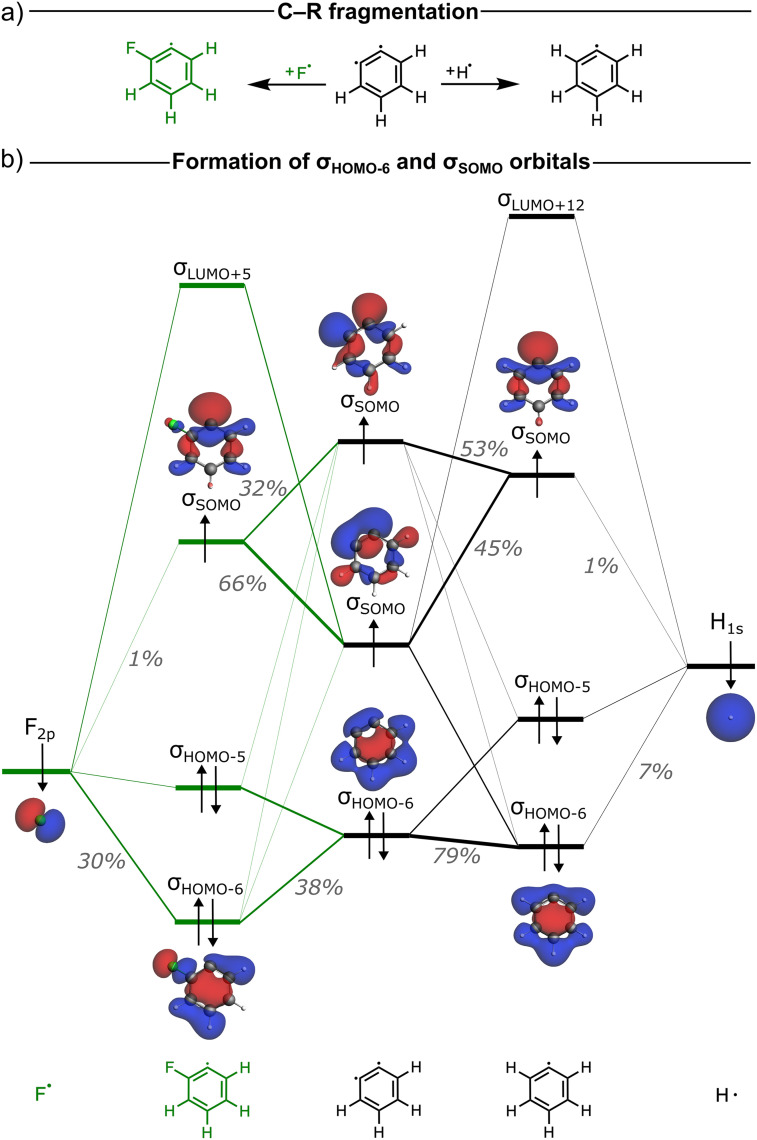
(a) Fragmentation of the C–R bond (R = F *versus* H). (b) Qualitative MO diagram showing the formation of the *σ*_SOMO_ and *σ*_HOMO−6_ of the aryl radical 
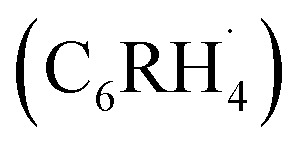
 from the interaction of 
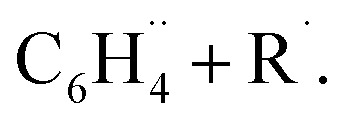
 SFO gross Mulliken contributions (in gray) are shown only for *σ*_SOMO_ and *σ*_HOMO−6_; for details on the other orbitals, see Fig. S8 and Table S4. Computed at ZORA-BLYP-D3(BJ)/TZ2P.

The essential question remaining is: why does fluorination reduce the Pauli-repulsive overlap between the occupied orbitals on the 
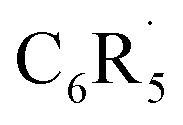
 fragment and the radical electron of the hydrogen atom and thus strengthen the aryl C–H bond? To address this question, we analyze the formation of the aryl fragment 
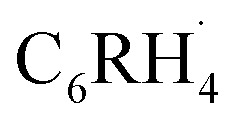
 in benzene (R = H) and fluorobenzene (R = F) from the 
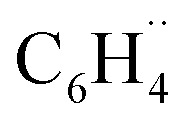
 biradical and R˙ (see Fig. 5a). This approach enables us to understand how the relevant 
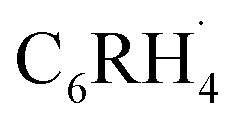
 orbitals arise from the same 
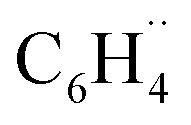
 biradical, and how their nature becomes different in the case of a fluorine substituent R = F in fluorobenzene *versus* a hydrogen substituent R = H in benzene. The resulting MO diagram is shown qualitatively in Fig. 5b (for details, see Fig. S10–S12 and Tables S4–S6). From this point onward, we focus on the *ortho* position, where the reduction of Pauli repulsion is most pronounced and where the proximity of the substituent would intuitively suggest a repulsive effect.

The *σ*_HOMO−6_ orbitals of the 
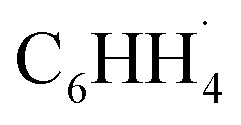
 and 
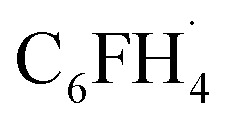
 radicals arise from a σ-bonding interaction between a doubly occupied σ-orbital of the 
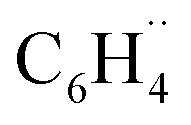
 biradical and the singly occupied hydrogen 1s (H_1s_) or fluorine 2p (F_2p_) orbitals, respectively (see Fig. 5b). The F_2p_ orbital lies lower in energy than the H_1s_ orbital because fluorine is more electronegative than hydrogen. As a result, the F_2p_ orbital is closer in energy to the doubly occupied σ-orbital of the 
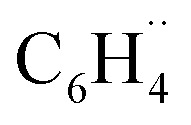
 fragment than the H_1s_ orbital, which leads to a larger fluorine contribution to the formation of the bonding combination, which is the *σ*_HOMO−6_ orbital (see gross Mulliken contributions in Fig. 5b). This greater contribution of fluorine polarizes the *σ*_HOMO−6_ orbital towards the fluorine atom and away from the carbon radical. Consequently, the overlap between *σ*_HOMO−6_ and the hydrogen 1s orbital across the aromatic C–H bond between the carbon-radical center of 
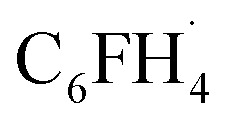
 and H˙ is reduced. This is a clear manifestation of fluorine's inductive effect, which predominantly affects the σ-system in fluorobenzene, as previously discussed elsewhere.^28^ Furthermore, note that neither the F_2p_ nor the H_1s_ contributes significantly to the formation of the *σ*_SOMO_ in the aryl radical. As a result, the *σ*_SOMO_ is not polarized by the substituent, leading to similar 〈H_1s_|*σ*_SOMO_〉 orbital overlaps and, consequently, similar electron-pair bond Δ*E*_pb_ and orbital interaction Δ*E*_oi_ energies for benzene and fluorobenzene (see Fig. S9 for overlaps and Table S7 for Δ*E*_pb_ values).^29^

### Predictive power of the model

Finally, we wish to extend our model to related systems. To this end, we have extended our MO and EDA analyses of the C–H bonds to a broader set of monosubstituted benzenes C_6_H_5_R with R still including H and F, but now also Cl, Br, I, and Li. Proceeding from the model we have established, one would expect that electronegative substituents R strengthen C–H bonds, unless, in cases of adjacent C–H bonds, R becomes so big that it destabilizes this C–H bond through direct R⋯H Pauli repulsion.^5^ On the other hand, electropositive substituents R are then expected to weaken the C–H bonds because they polarize orbital amplitude toward the pertinent carbon atoms. The results confirm this expectation.

**Fig. 6:**
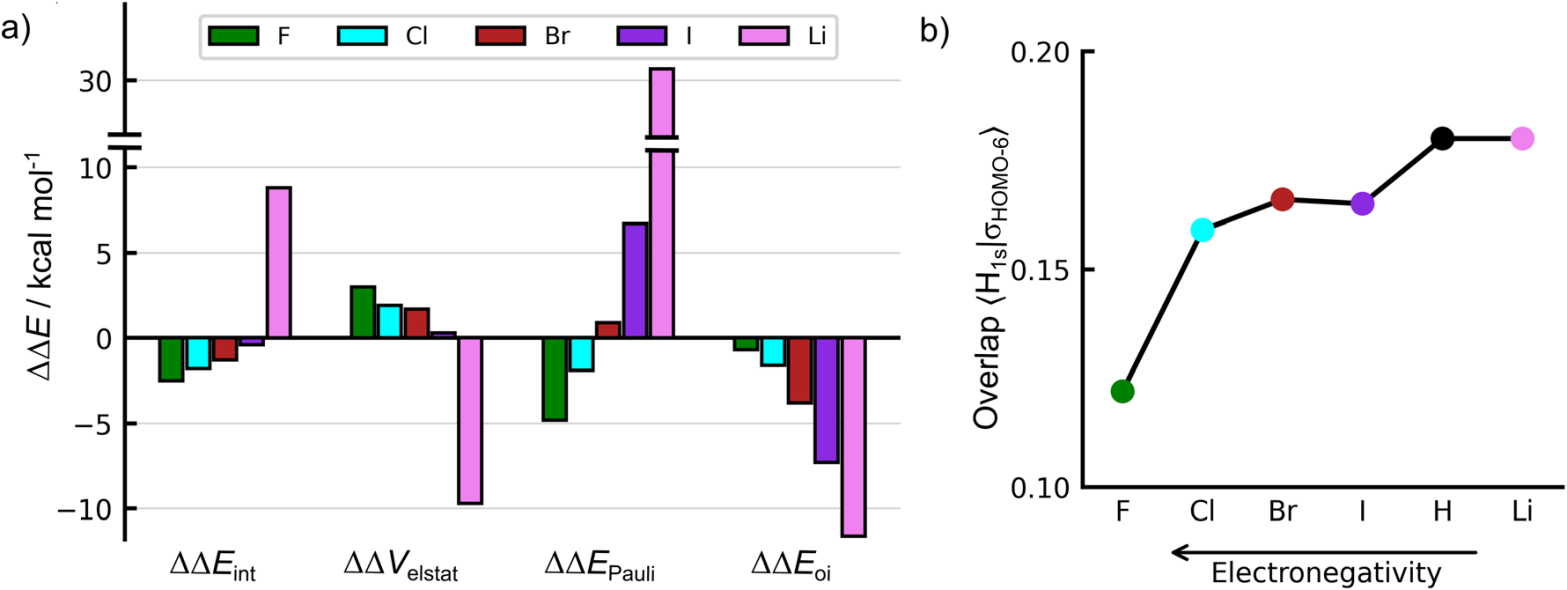
(a) Energy decomposition analysis of the *ortho*-C–H bond in mono-substituted benzenes C_6_RH_5_ (R = F, Cl, Br, I, and Li) relative to benzene at a consistent geometry with a C–H distance of 1.088 Å, and (b) the associated repulsive overlap 〈H_1s_|*σ*_HOMO−6_〉. Computed at ZORA-BLYP-D3(BJ)/TZ2P.

The bond dissociation enthalpies Δ*H*, bond dissociation energies Δ*E*, together with the ASM and EDA terms for this series of monosubstituted benzenes, are given in Table S7. The C–H bond dissociation enthalpy is larger than that in benzene for C_6_FH_5_ (−111.8 kcal mol^−1^) and C_6_ClH_5_ (−110.9 kcal mol^−1^) but very close to that in benzene (−109.3 kcal mol^−1^) for the other halogens (Br, −110.3; I, −109.4 kcal mol^−1^). However, the C–H bond dissociation enthalpy is significantly smaller in C_6_LiH_5_ (−91.9 kcal mol^−1^). A comparison with experimental BDE data would be valuable; however, such data are not listed in ref. 19 for the systems with R = Cl, Br, I, and Li. As expected, the Δ*H* values follow the same trends as the bond energies Δ*E*, which are in turn dominated by the interaction energy Δ*E*_int_. The relative EDA terms for the C–H bonds in the C_6_RH_5_ (R = F, Cl, Br, I, Li) systems are depicted in Fig. 6a. As shown in Fig. 6b, the inductive effect responsible for the reduction in Pauli repulsion in fluorobenzene is also observed in the other halobenzenes. In contrast to the case of fluorine, the orbital term is of the same order of magnitude as the Pauli repulsion term for Cl, Br, and I. Thus, the increase in Δ*E*_oi_ compensates for the variation in the Δ*E*_Pauli_ and results in values of Δ*E*_int_ for Cl, Br, and I within 1.4 kcal mol^−1^ of one another (Fig. 6a and Table S7).

As in the case of fluorine, strong contributions to the decrease of the Pauli repulsion are found in *σ*_HOMO−6_. The halogen substituent polarizes the *σ*_HOMO−6_ orbital toward itself and away from the C–H bonding region, leading to a progressive decrease in 〈H_1s_|*σ*_HOMO−6_〉 overlap as the electronegativity of the substituent increases from I to F. However, the EDA results in Fig. 6a (the complete dataset is provided in Table S7) reveal that inductive effects alone cannot fully account for the observed trends in Pauli repulsion across the halobenzene series. Although bromine and iodine are more electronegative than hydrogen and thus exert a higher inductive effect, the Pauli repulsion is actually higher in bromobenzene and iodobenzene compared to benzene. This apparent discrepancy arises from the increase in size of the substituent atom. As the size of R increases, the spatial extension of the valence AOs and the number of subvalence shells increase, causing a stronger Pauli repulsion. Thus, the observed trends in Pauli repulsion reflect a balance between two opposing effects, namely, the electron-withdrawing (inductive) effect, which reduces Pauli repulsion, and the steric (size-related) effect, which enhances Pauli repulsion.

For fluorine and chlorine, the most electronegative atoms in the series, the atom size effect is offset by the electron-withdrawing (inductive) effect, resulting in reduced 〈H_1s_|*σ*_HOMO−6_〉 overlap (see Fig. 6b), negative ΔΔ*E*_Pauli_, and stronger C–H bonds compared to benzene. In the case of bromine, the atom size effect begins to outweigh the inductive effect, leading to a modest increase in Pauli repulsion (ΔΔ*E*_Pauli_ = +0.9 kcal mol^−1^). Nevertheless, this destabilization is compensated by an enhanced orbital interaction,^30^ still resulting in a stronger *ortho*-C–H bond compared to benzene. For iodobenzene, the atom size effect becomes dominant, resulting in greater Pauli repulsion. However, even in this case, the gain in attractive orbital interactions counterbalances the increase in Δ*E*_Pauli_, yielding a C–H bond with nearly the same strength as in benzene.

**Fig. 7:**
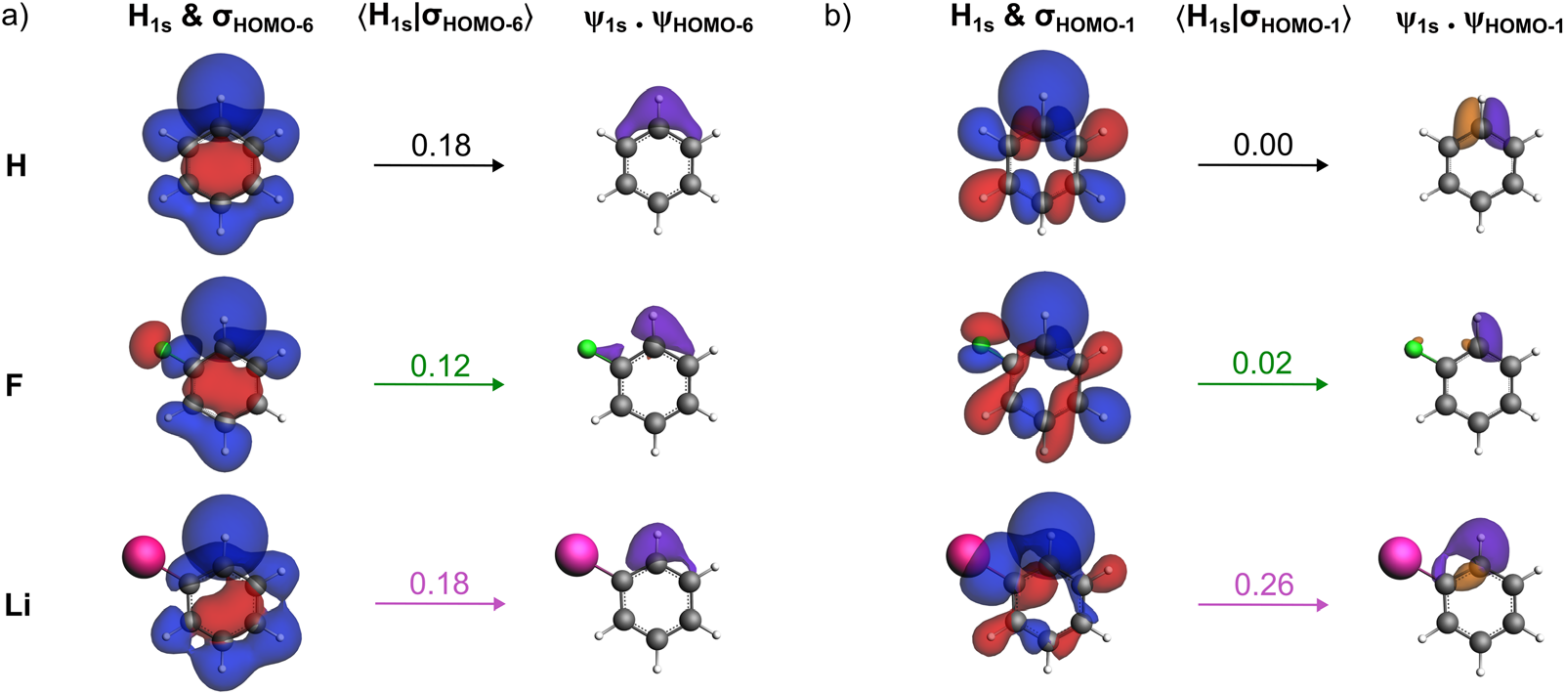
For the model systems with R = H, F, and Li: (a) H_1s_ AO and C_6_H_4_R˙ *σ*_HOMO−6_ (isovalue = 0.03 au) at their position in C_6_H_4_R–H, 〈H_1s_|*σ*_HOMO−6_〉 orbital overlap, and overlap density given by the product of fragment orbitals *ψ*_1s_·*ψ*_HOMO−6_ (isovalue = 0.002 au). (b) H_1s_ AO and C_6_H_4_R˙ *σ*_HOMO−1_ (isovalue = 0.03 au) at their position in C_6_H_4_R–H, 〈H_1s_|*σ*_HOMO−1_〉 orbital overlap, and overlap density given by the product of fragment orbitals *ψ*_1s_·*ψ*_HOMO−1_ (isovalue = 0.002 au). Computed at ZORA-BLYP-D3(BJ)/TZ2P.

Finally, we have also investigated phenyllithium (C_6_H_5_Li), a system in which the substituent R is less electronegative than hydrogen. The 2s orbital of lithium lies significantly higher in energy than the np orbitals of the halogens and does not contribute meaningfully to the formation of the *σ*_HOMO−6_ orbital in the aryl radical. As a result, the 〈H_1s_|*σ*_HOMO−6_〉 orbital overlap in phenyllithium is nearly identical to that in benzene (see Fig. 7a). However, lithium does affect higher-energy orbitals of the aryl fragment. Notably, Fig. 7b shows a substantial increase in the 〈H_1s_|*σ*_HOMO−1_〉 orbital overlap in C_6_H_4_Li–H relative to both benzene and fluorobenzene. Unlike the halogens, lithium raises the orbital amplitude in the C–H bonding region rather than depleting it, effectively donating electron density into the carbon scaffold of C–C σ bonds through an effect opposite to that of fluorine. This electron-donating character leads to increased occupied–occupied overlap and thus increased Pauli repulsion across the aromatic C–H bond. This is reminiscent of the lone-pair shielded radical effect induced by the substituent on the C–C bond strength.^31^ This effect outweighs all stabilizing contributions, resulting in a substantially weaker C–H bond.

To disentangle size and inductive effects, we also performed EDA analyses for the same substituents at the *para* position. In this configuration, direct R⋯H repulsion is eliminated, allowing the intrinsic electronic nature of the substituent (electron-withdrawing or electron-donating) to dominate the Pauli repulsion trends. As shown in Fig. S13, all substituents more electron-withdrawing than hydrogen lead to a decrease in Pauli repulsion, following the electronegativity trend. In contrast, for phenyllithium, the only electron-donating substituent, Pauli repulsion remains significantly higher than in benzene, even in the absence of direct steric interactions.

## Conclusion

Fluorine substitution strengthens the C–H bond in fluorinated benzenes C_6_R_5_H (R = H, F), as indicated by the increase in the homolytic bond dissociation energies (BDE). Our quantum chemical bonding analyses, based on dispersion-corrected, (scalar) relativistic density functional theory, uncover that fluorination strengthens the C–H bond in large part by reducing steric (Pauli) repulsion between filled orbitals. As demonstrated previously, the effects of multiple fluorine substitution are additive and markedly greater for *ortho* than for *para* substitution while the effect of *meta* substitution is barely significant. The *ortho* effect on BDE is calculated to be *ca.* 2.9 times the effect of *para* substitution. The EDA analysis shows that the Pauli repulsion is an important contributor to the bond strengthening but that this *ortho*/*para* ratio can be reproduced by the sum of the Pauli repulsion and orbital terms.

Our quantitative MO and energy decomposition analyses reveal how the strong inductive effect of the fluorine atom withdraws electron density, especially from the ipso carbon of the aryl fragment and polarizes its closed-shell molecular orbitals towards the fluorine atom. This polarization reduces the spatial extension of the occupied orbitals on the aryl fragment towards the hydrogen 1s orbital. Therefore, the same-spin orbital overlap across the C–H bond shrinks, and Pauli repulsion becomes weaker, resulting in stronger C–H bonds.

Extension of the analysis to other monosubstituted benzenes C_6_RH_5_ (R = Cl, Br, I, Li) shows that substituents R modulate the strength of the adjacent C–H bond by reducing or enhancing Pauli repulsion, either *via* an indirect, inductive effect or *via* direct steric repulsion between C–R and C–H bonds. For R = F, the C–H BDE is notably bigger than for other halogens which, in turn, are slightly bigger than that of benzene. For R = F, the strong inductive effects decrease the overall Pauli repulsion, resulting in a stronger C–H bond. For R = Cl, the overall Pauli repulsion remains smaller than for benzene. In contrast, for R = Br and I, weaker inductive effects are offset by larger atomic size, which increases direct R⋯H steric repulsion. For all halogens except fluorine, this repulsion is balanced by enhanced orbital attraction, yielding C–H bonds of similar strength for R = Cl, Br, I, and H. Thus, fluorine remains as a special case among electron-withdrawing substituents that we have studied so far. Finally, for R = Li, the inductive effect is inverted: lithium pushes electron density to the aryl fragment, increasing Pauli repulsion and leading to the weakest C–H bond in the series.

## Author contributions

RNP, OE, and FMB conceived the project, which was supervised by DRS, AACB, and CFG. DASO carried out the quantum-chemical computations and bonding analyses and drafted the manuscript. All authors discussed the results and reviewed the manuscript.

## Conflicts of interest

There are no conflicts of interest to declare.

## Supplementary Material

SC-OLF-D6SC01846G-s001

## Data Availability

All data of this study are available in the main text and supplementary information (SI). Supplementary information is available. See DOI: https://doi.org/10.1039/d6sc01846g.
