# Supplementary material for: How fluorine substituents strengthen aryl C–H bonds
Source: Chem Sci. 2026 Apr 27;17(23):11390–402. doi: 10.1039/d6sc01846g (PMC13133636; doi:10.1039/d6sc01846g)
Supplement: SC-017-D6SC01846G-s001 [file SC-017-D6SC01846G-s001.pdf]

Supplementary Information (SI) for Chemical Science.
This journal is © The Royal Society of Chemistry 2026

Supporting Information for

# How Fluorine Substituents Strengthen Aryl C–H Bonds

Daniel A. Santos Oliveira,[a,b] Daniela Rodrigues Silva,[a] Ataualpa A. C. Braga,[b] Célia Fonseca Guerra,[a] Robin N. Perutz,*[c] Odile Eisenstein,*[d,e] and F. Matthias Bickelhaupt*[a,f,g]

a Department of Chemistry and Pharmaceutical Sciences, AIMMS, Vrije Universiteit Amsterdam, De Boelelaan 1108, 1081 HZ Amsterdam, The Netherlands.
E–mail: f.m.bickelhaupt@vu.nl
Web address: https://www.theochem.nl

b Department of Fundamental Chemistry, Institute of Chemistry, University of São Paulo, Av. Prof. Lineu Prestes, 748, São Paulo, 055508-000, Brazil.

c Department of Chemistry, University of York, York, YO10 5DD, U.K.
E–mail: robin.perutz@york.ac.uk

d ICGM, Univ. Montpellier, CNRS, ENSCM, Montpellier, France.
E–mail: odile.eisenstein@umontpellier.fr

e Department of Chemistry and Hylleraas Centre for Quantum Molecular Sciences, University of Oslo, PO Box 1033 Blindern, 0315 Oslo, Norway.

f Institute of Molecules and Materials, Radboud University, Heyendaalseweg 135, 6525 AJ Nijmegen, The Netherlands.

g Department of Chemical Sciences, University of Johannesburg, Auckland Park, Johannesburg 2006, South Africa.

## Table of Contents

**Table S1**. Energy decomposition analysis terms (in kcal mol$^{-1}$) of the C–H bond for substituted benzenes at the equilibrium geometry.

**Table S2**. Energy decomposition analysis terms (in kcal mol$^{-1}$) of the C–H bond for substituted benzenes at a consistent geometry with a C–H distance of 1.088 Å.

**Figure S1**. Linear correlation between $\Delta\Delta H$ and the C–H bond lengths. Computed at ZORA-BLYP-D3(BJ)/TZ2P.

**Figure S2**. Multiple linear regression relating the number of fluorine substituents at the *ortho*, *meta*, and *para* positions to relative a) C–H bond enthalpies $\Delta\Delta H$ and b) C–H bond energies $\Delta\Delta E$.

**Figure S3**. Subset of fluorinated benzenes $C_6R_5H$ (R = H, F) used in both analyses at the consistent-geometry (Table S2) and as a function the C–H bond distance (Figure S6 and S7).

**Figure S4**. Energy decomposition analysis terms of the C–H bonds in polyfluorinated benzenes relative to benzene at a consistent geometry with a C–H distance of 1.088 Å. Computed at ZORA-BLYP-D3(BJ)/TZ2P.

**Figure S5**. Energy decomposition analysis terms for the C–H bond, using four different reference systems: a) benzene, b) the *ortho*-C–H bond in fluorobenzene, c) the *meta*-C–H bond in fluorobenzene, and d) the adjacent C–H bond in 1,2-difluoro-benzene. For each reference system, a fluorine substituent was introduced at the *ortho*, *meta*, and *para* positions, and the corresponding relative EDA values were computed at ZORA-BLYP-D3(BJ)/TZ2P.

**Figure S6**. C–H bond enthalpies (colored and gray bars), referenced to the corresponding monosubstituted values (indicated by the vertical lines within the bars), for (a) fluorobenzene and (b) polyfluorinated benzenes. Pauli repulsion terms (colored and gray bars), referenced to the corresponding monosubstituted values, for (c) fluorobenzene and (d) polyfluorinated benzenes. C–H bond enthalpies (colored and gray bars), referenced to the regression coefficients, for (e) fluorobenzene and (f) polyfluorinated benzenes. Pauli repulsion terms (colored and gray bars), referenced to the regression coefficients, for (g) fluorobenzene and (h) polyfluorinated benzenes. Computed at ZORA-BLYP-D3(BJ)/TZ2P. All values relative to those in benzene.

**Figure S7**. Energy decomposition analysis terms as a function of the C–H bond distance in benzene and fluorobenzene. The dashed vertical line indicates the consistent geometry with a C–H bond distance of 1.088 Å. Computed at ZORA-BLYP-D3(BJ)/TZ2P.

**Figure S8**. Energy decomposition analysis terms as a function of the C–H bond distance in benzene and the polyfluorinated benzenes. The dashed vertical line indicates the consistent geometry with a C–H bond distance of 1.088 Å. Computed at ZORA-BLYP-D3(BJ)/TZ2P.

**Table S3**. Orbital overlaps *S* between the hydrogen 1s orbital and the orbitals in the σ-framework in the aryl fragment, along with their variation relative to benzene ($\Delta S$). Only orbital overlaps with nonzero values in benzene (**B**) are shown. Computed at ZORA-BLYP-D3(BJ)/TZ2P.

**Figure S9**. Orbital overlaps as a function of the C–H bond distance in benzene and fluorobenzene computed at ZORA-BLYP-D3(BJ)/TZ2P.

**Figure S10**. Qualitative MO diagram showing the formation of the $\sigma_{SOMO}$ and $\sigma_{HOMO-6}$ of the *ortho*-aryl radical ($C_6H_4R^{•}$) from the interaction of $C_6H_4^{••}$ + $R^{•}$ (R = F *versus* H). The orbital energies (in eV) are given in red, gross population (in electrons) in blue (R = F) and black (R = H), and SFO Gross Mulliken contributions in gray. Computed at ZORA-BLYP-D3(BJ)/TZ2P.

**Table S4.** SFO Gross Mulliken contributions (%) to the MOs of the *ortho*-aryl radical ($C_6H_4R^•$) formed from the interaction of $C_6H_4^{••}$ + $R^•$ (R = F or H). Computed at ZORA-BLYP-D3(BJ)/TZ2P.

**Figure S11**. Qualitative MO diagram showing the formation of the $\sigma_{SOMO}$ and $\sigma_{HOMO-6}$ of the *meta*-aryl radical ($C_6H_4R^•$) from the interaction of $C_6H_4^{••}$ + $R^•$ (R = F *versus* H). The orbital energies (in eV) are given in red, gross population (in electrons) in blue (R = F) and black (R = H), and SFO Gross Mulliken contributions in gray. Computed at ZORA-BLYP-D3(BJ)/TZ2P.

**Table S5.** SFO Gross Mulliken contributions (%) to the MOs of the *meta*-aryl radical ($C_6H_4R^•$) formed from the interaction of $C_6H_4^{••}$ + $R^•$ (R = F or H). Computed at ZORA-BLYP-D3(BJ)/TZ2P.

**Figure S12**. Qualitative MO diagram showing the formation of the $\sigma_{SOMO}$ and $\sigma_{HOMO-6}$ of the *para*-aryl radical ($C_6H_4R^•$) from the interaction of $C_6H_4^{••}$ + $R^•$ (R = F *versus* H). The orbital energies (in eV) are given in red, gross population (in electrons) in blue (R = F) and black (R = H), and SFO Gross Mulliken contributions in gray. Computed at ZORA-BLYP-D3(BJ)/TZ2P.

**Table S6.** SFO Gross Mulliken contributions (%) to the MOs of the *para*-aryl radical ($C_6H_4R^•$) formed from the interaction of $C_6H_4^{••}$ + $R^•$ (R = F or H). Computed at ZORA-BLYP-D3(BJ)/TZ2P.

**Table S7**. Energy decomposition analysis (in kcal $mol^{-1}$) of the *ortho*-C–H bond in mono-substituted benzenes $C_6H_5R$ (R= H, F, Cl, Br, I, and Li) at a consistent geometry with a C–H distance of 1.088 Å. Computed at ZORA-BLYP-D3(BJ)/TZ2P.

**Figure S13**. Energy decomposition analysis of the *para*-C–H bond in mono-substituted benzenes $C_6RH_5$ (R = F, Cl, Br, I, and Li) relative to benzene at a consistent geometry with a C–H distance of 1.088 Å. Computed at ZORA-BLYP-D3(BJ)/TZ2P.

**Table S8**. Cartesian coordinates (in Å), energies (electronic energy E and enthalpy *H*, in kcal $mol^{-1}$), number of imaginary frequencies ($N_{imag}$), and total spin number (*S*) of the equilibrium geometries of all systems studied herein, computed at ZORA-BLYP-D3(BJ)/TZ2P.

**Table S1.** Energy decomposition analysis terms (in kcal mol$^{-1}$) of the C–H bond for the complete set of substituted benzenes at the equilibrium geometry.[a]

| Species | $\Delta H$ | $\Delta\Delta H$ | $\Delta\Delta H^{b}$ | $\Delta E$ | $\Delta E_{\mathrm{strain}}$ | $\Delta E_{\mathrm{int}}$ | $\Delta V_{\mathrm{elstat}}$ | $\Delta E_{\mathrm{Pauli}}$ | $\Delta E_{\mathrm{oi}}$ | $\Delta E_{\mathrm{disp}}$ | $\Delta E_{\mathrm{spinpol}}$ |
|---|---|---|---|---|---|---|---|---|---|---|---|
| **B** | –109.3[c] | 0.0 | 0.0 | –115.9 | 1.8 | –117.7 | –64.9 | 94.9 | –149.5 | –0.9 | 2.7 |
| **o** | –111.8 | –2.5 | –2.6 | –118.4 | 1.8 | –120.2 | –62.0 | 90.4 | –150.5 | –0.9 | 2.8 |
| **m** | –109.7 | –0.4 | –0.2 | –116.3 | 1.8 | –118.1 | –64.1 | 94.6 | –150.4 | –0.9 | 2.7 |
| **p** | –110.6 | –1.3 | –1.1 | –117.2 | 1.7 | –118.9 | –63.5 | 91.1 | –148.2 | –1.0 | 2.7 |
| **o-m** | –111.8 | –2.5 | –2.5 | –118.3 | 1.9 | –120.2 | –61.1 | 90.3 | –151.3 | –0.9 | 2.8 |
| **m-p** | –110.7 | –1.4 | –1.1 | –117.3 | 1.8 | –119.1 | –63.2 | 91.8 | –149.5 | –0.9 | 2.7 |
| **2o** | –114.8 | –5.5 | –5.3 | –121.1 | 1.8 | –122.9 | –58.8 | 84.3 | –150.3 | –0.9 | 2.8 |
| **2m** | –110.2 | –0.9 | –0.5 | –116.8 | 1.9 | –118.7 | –63.0 | 93.6 | –151.0 | –1.0 | 2.7 |
| **o-p** | –113.0 | –3.7 | –3.5 | –119.5 | 1.7 | –121.2 | –60.7 | 87.0 | –149.3 | –1.0 | 2.8 |
| **o-m'** | –112.2 | –2.9 | –2.7 | –118.7 | 1.8 | –120.5 | –61.5 | 90.7 | –151.6 | –0.9 | 2.8 |
| **o-m-p** | –112.7 | –3.4 | –3.3 | –119.2 | 1.9 | –121.1 | –60.2 | 87.8 | –150.5 | –1.0 | 2.8 |
| **2m-p** | –110.9 | –1.6 | –1.2 | –117.5 | 1.9 | –119.4 | –62.4 | 91.7 | –150.5 | –0.9 | 2.7 |
| **2o-m** | –114.7 | –5.4 | –5.3 | –121.1 | 1.8 | –122.9 | –58.4 | 85.0 | –151.5 | –0.9 | 2.9 |
| **o-2m** | –112.4 | –3.1 | –2.8 | –118.9 | 1.9 | –120.8 | –60.2 | 89.4 | –151.8 | –1.0 | 2.8 |
| **o-m-p'** | –113.1 | –3.8 | –3.6 | –119.6 | 1.8 | –121.4 | –60.5 | 88.0 | –150.7 | –1.0 | 2.8 |
| **2o-p** | –115.7 | –6.4 | –6.1 | –122.1 | 1.7 | –123.8 | –57.6 | 81.3 | –149.2 | –1.0 | 2.7 |
| **o-2m-p** | –113.0[c] | –3.7 | –3.5 | –119.5 | 1.9 | –121.4 | –59.5 | 87.8 | –151.6 | –1.0 | 2.9 |
| **2o-2m** | –115.0 | –5.7 | –5.4 | –121.3 | 1.9 | –123.2 | –57.2 | 83.7 | –151.6 | –1.0 | 2.9 |
| **2o-m-p** | –115.5 | –6.2 | –6.0 | –121.8 | 1.8 | –123.6 | –57.4 | 82.7 | –150.8 | –1.0 | 2.9 |
| **2o-2m-p** | –115.5[c] | –6.2 | –6.0 | –121.8 | 1.9 | –123.7 | –56.4 | 82.4 | –151.6 | –1.0 | 2.9 |

[a] Computed at ZORA-BLYP-D3(BJ)/TZ2P level of theory. [b] Relative C–H bond enthalpies reported in reference 4a and computed at B3PW91/6-31G(d,p) level of theory. [c] Experimental values for **B**, **o-2m-p**, and **2o-2m-p** are –113.2, –115.0, and –116.5 kcal mol$^{-1}$, respectively.[27]

**Table S2.** Energy decomposition analysis terms (in kcal mol$^{-1}$) of the C–H bond for substituted benzenes at a consistent geometry[a] with a C–H distance of 1.088 Å.[b] Energies relative to benzene (**B**) for polyfluorinated benzenes are shown in Figure S2.

| Species | $\Delta E_{int}$ | $\Delta V_{elstat}$ | $\Delta E_{Pauli}$ | $\Delta E_{oi}$ | $\Delta E_{oi,\sigma}$ | $\Delta E_{oi,\pi}$ | $\Delta E_{disp}$ | $\Delta E_{spinpol}$ |
|---|---|---|---|---|---|---|---|---|
| **B** | –117.7 | –64.9 | 94.9 | –149.5 | –148.0 | –1.6 | –0.9 | 2.7 |
| **o** | –120.2 | –61.9 | 90.1 | –150.2 | –148.8 | –1.4 | –1.0 | 2.8 |
| **m** | –118.1 | –64.1 | 94.5 | –150.3 | –148.9 | –1.5 | –0.9 | 2.7 |
| **p** | –118.9 | –63.5 | 90.8 | –148.0 | –146.6 | –1.4 | –0.9 | 2.7 |
| **2o** | –122.9 | –58.7 | 83.6 | –149.7 | –148.6 | –1.1 | –0.9 | 2.8 |
| **2m** | –118.7 | –63.0 | 93.3 | –150.8 | –149.4 | –1.4 | –0.9 | 2.7 |
| **2o-p** | –123.8 | –57.5 | 80.5 | –148.5 | –147.5 | –1.0 | –1.0 | 2.7 |
| **2m-p** | –119.4 | –62.3 | 91.2 | –150.1 | –148.8 | –1.3 | –0.9 | 2.7 |
| **2o-2m** | –123.2 | –57.1 | 83.1 | –151.1 | –150.2 | –1.0 | –1.0 | 2.9 |
| **2o-2m-p** | –123.7 | –56.3 | 81.8 | –151.0 | –150.1 | –0.9 | –1.0 | 2.8 |

[a] The C–H equilibrium distance for benzene. [b] Computed at ZORA-BLYP-D3(BJ)/TZ2P level of theory.

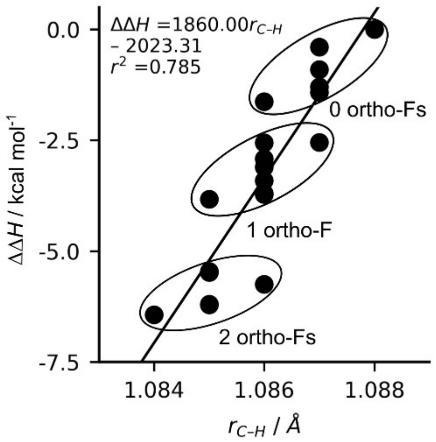


**Figure S1.** Linear correlation between $\Delta\Delta H$ and the C–H bond lengths. Computed at ZORA-BLYP-D3(BJ)/TZ2P.

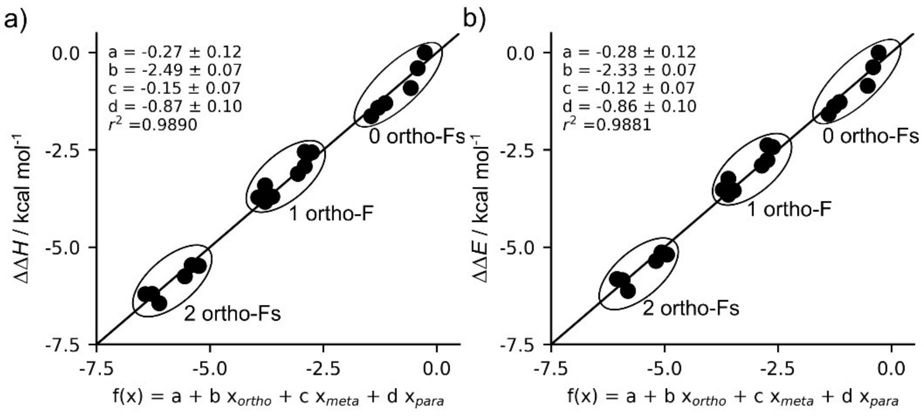


**Figure S2.** Multiple linear regression relating the number of fluorine substituents at the *ortho*, *meta*, and *para* positions to relative a) C–H bond enthalpies ΔΔ$H$ and b) C–H bond energies ΔΔ$E$.

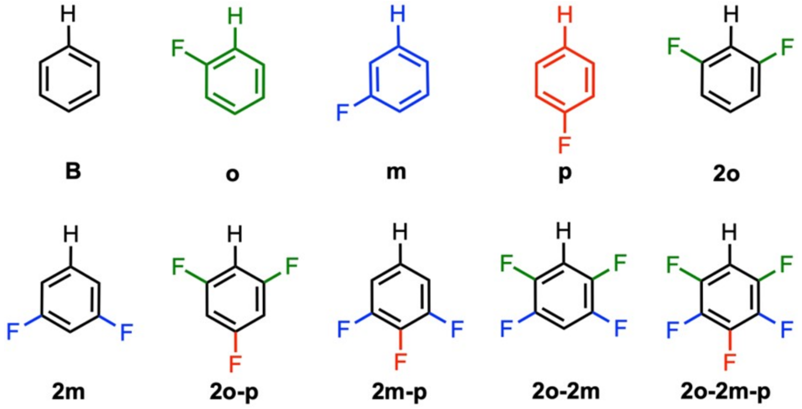


**Figure S3.** Subset of fluorinated benzenes $C_6R_5H$ (R = H, F) used in both analyses at the consistent-geometry (Table S2) and as a function of the C–H bond distance (Figure S5 and S6).

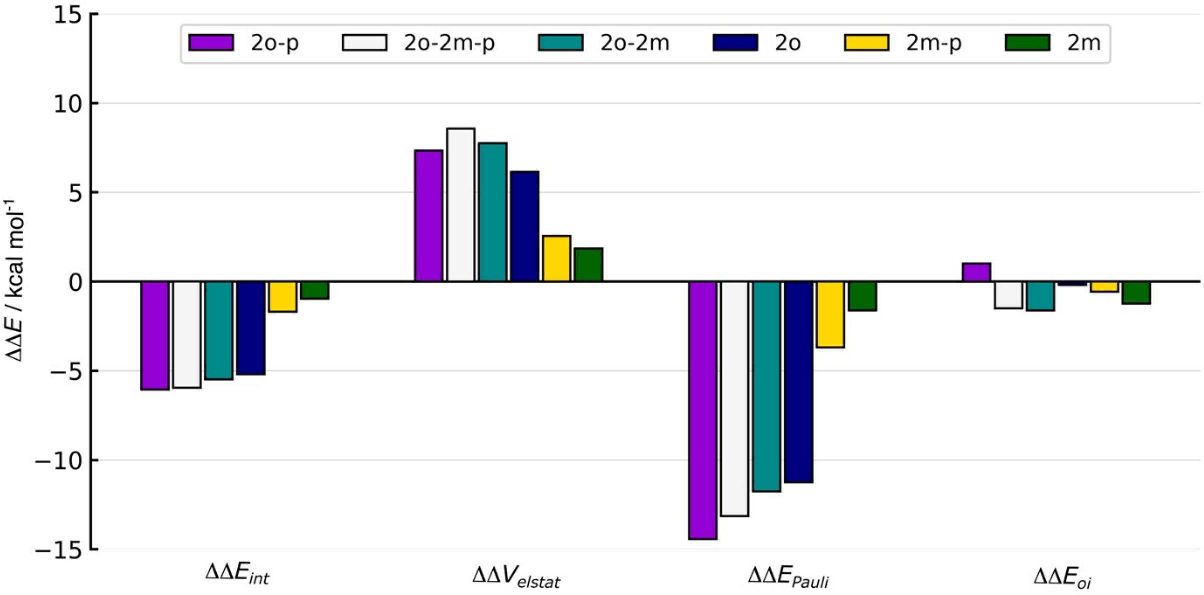


**Figure S4.** Energy decomposition analysis terms of the C–H bonds in polyfluorinated benzenes relative to benzene at a consistent geometry with a C–H distance of 1.088 Å. Computed at ZORA-BLYP-D3(BJ)/TZ2P.

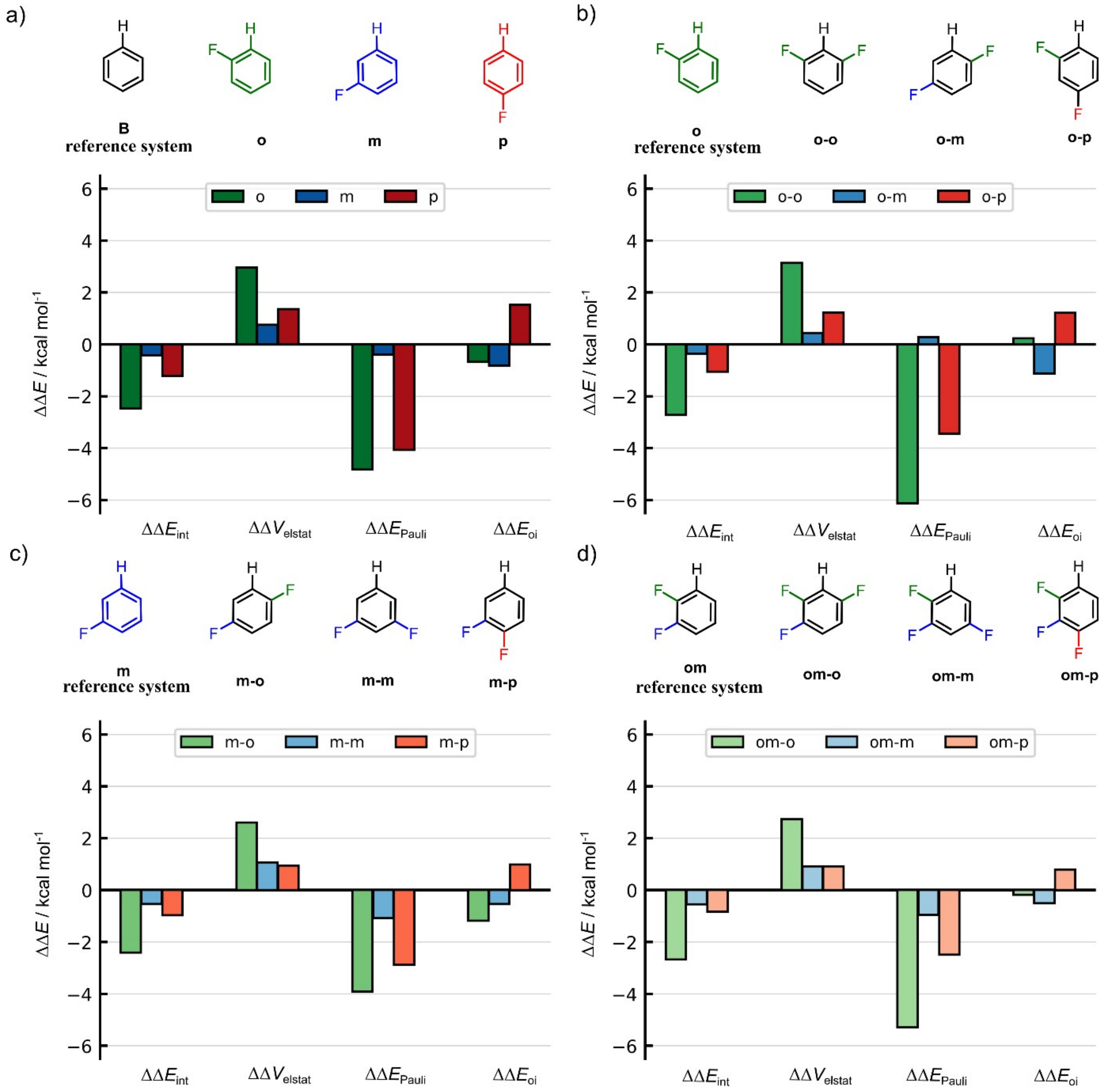


**Figure S5.** Energy decomposition analysis terms for the C–H bond, using four different reference systems: a) benzene, b) the *ortho*-C–H bond in fluorobenzene, c) the *meta*-C–H bond in fluorobenzene, and d) the adjacent C–H bond in 1,2-difluorobenzene. For each reference system, a fluorine substituent was introduced at the *ortho*, *meta*, and *para* positions, and the corresponding relative EDA values were computed at ZORA-BLYP-D3(BJ)/TZ2P.

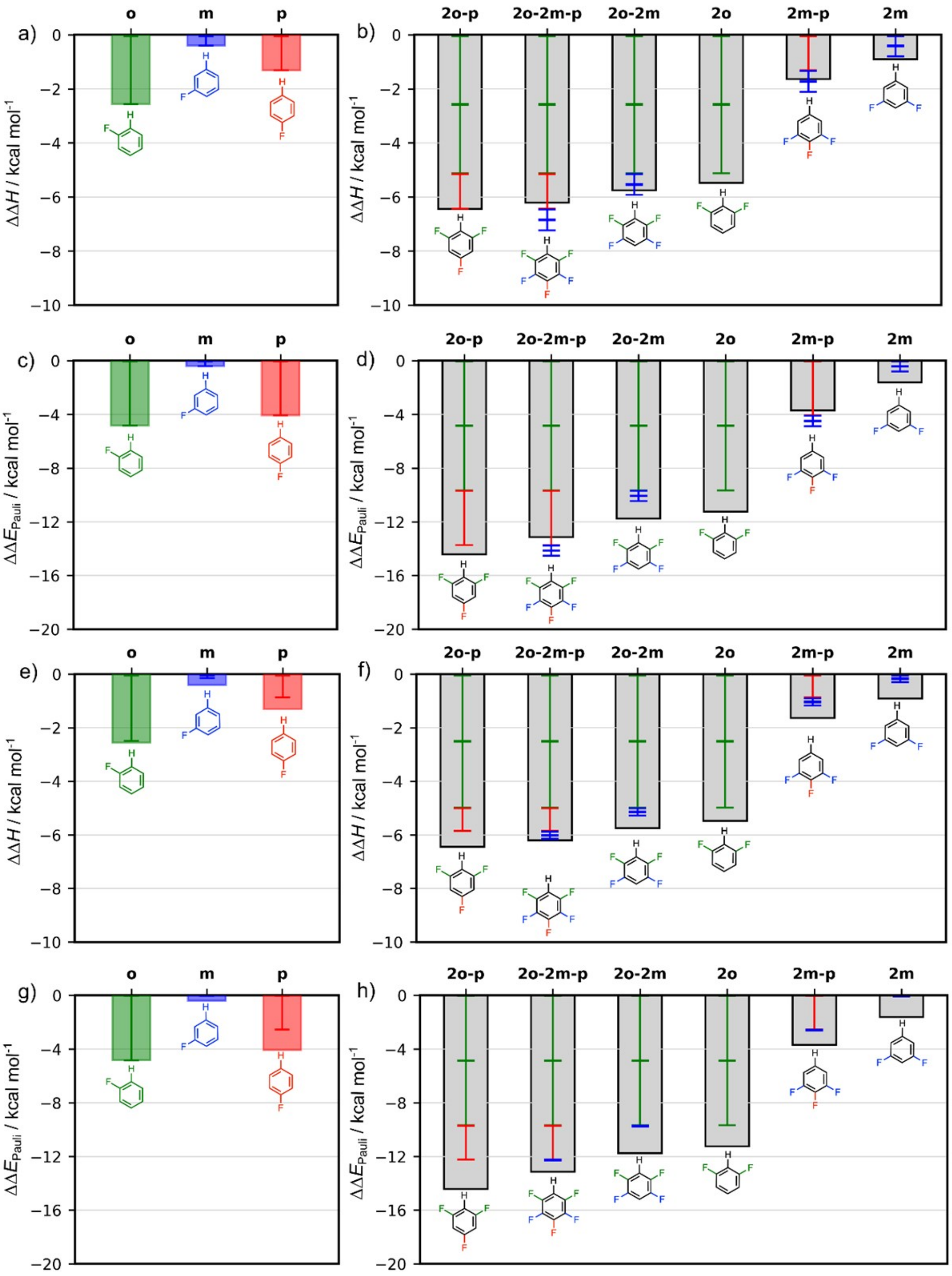


**Figure S6.** C–H bond enthalpies (colored and gray bars), referenced to the corresponding monosubstituted values (indicated by the vertical lines within the bars), for (a) fluorobenzene and (b) polyfluorinated benzenes. Pauli repulsion terms (colored and gray bars), referenced to the corresponding monosubstituted values, for (c) fluorobenzene and (d) polyfluorinated benzenes. C–H bond enthalpies (colored and gray bars), referenced to the regression coefficients, for (e) fluorobenzene and (f) polyfluorinated benzenes. Pauli repulsion terms (colored and gray bars), referenced to the regression coefficients, for (g) fluorobenzene and (h) polyfluorinated benzenes. Computed at ZORA-BLYP-D3(BJ)/TZ2P. All values relative to those in benzene.

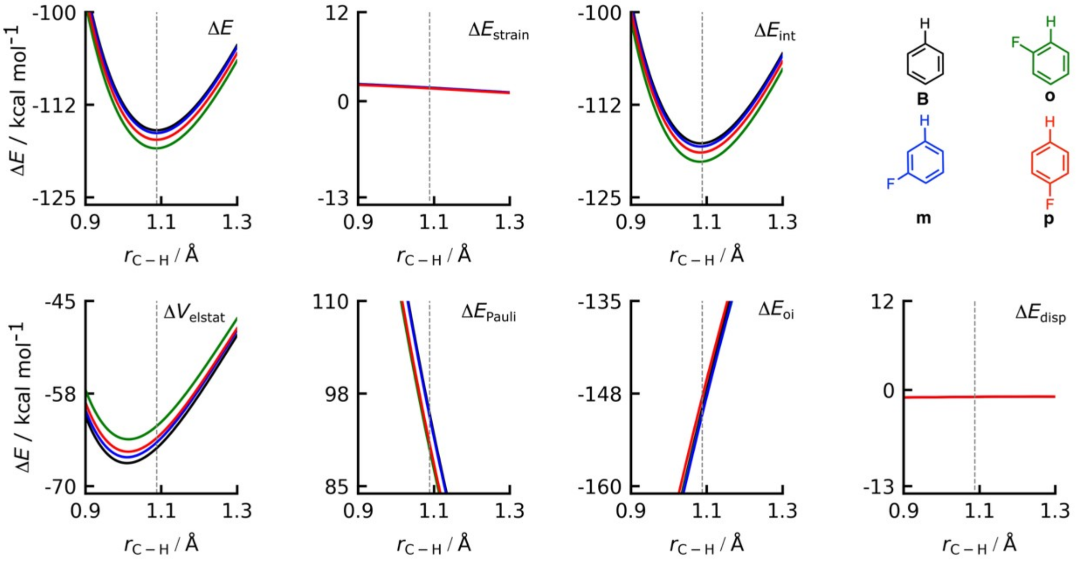


**Figure S7.** Energy decomposition analysis terms as a function of the C–H bond distance in benzene and fluorobenzene. The dashed vertical line indicates the consistent geometry with a C–H bond distance of 1.088 Å. Computed at ZORA-BLYP-D3(BJ)/TZ2P.

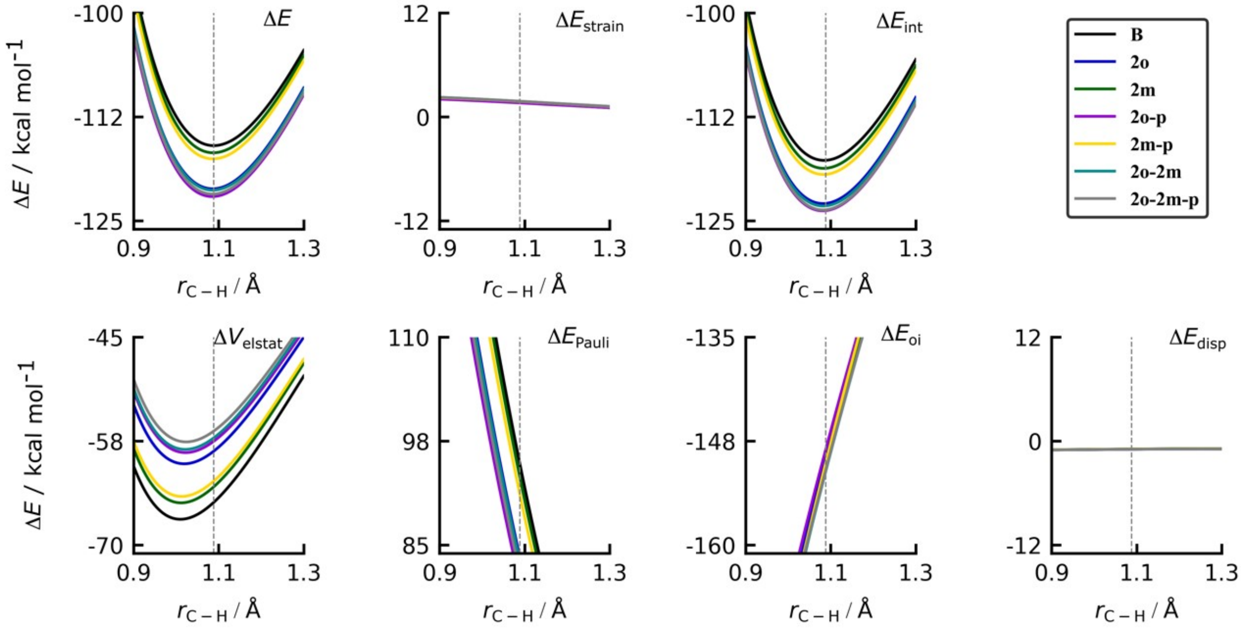


**Figure S8.** Energy decomposition analysis terms as a function of the C–H bond distance in benzene and the polyfluorinated benzenes. The dashed vertical line indicates the consistent geometry with a C–H bond distance of 1.088 Å. Computed at ZORA-BLYP-D3(BJ)/TZ2P.

**Table S3.** Orbital overlaps $S$ between the hydrogen 1s orbital and the orbitals in the σ- framework in the aryl fragment, along with their variation relative to benzene ($\Delta S$). Only orbital overlaps with nonzero values in benzene (**B**) are shown. Computed at ZORA-BLYP-D3(BJ)/TZ2P.

| Orbital | System | $S$ | $\Delta S$ | Orbital | System | $S$ | $\Delta S$ |
|---|---|---|---|---|---|---|---|
| $\sigma_{SOMO}$ | **B** | 0.61 | 0.00 | $\sigma_{HOMO-7}$ | **B** | 0.18 | 0.00 |
| | **o** | 0.61 | 0.00 | | **o** | 0.16 | –0.02 |
| | **m** | 0.60 | –0.01 | | **m** | 0.16 | –0.02 |
| | **p** | 0.61 | 0.00 | | **p** | 0.17 | –0.01 |
| $\sigma_{HOMO-2}$ | **B** | 0.21 | 0.00 | $\sigma_{HOMO-9}$ | **B** | 0.28 | 0.00 |
| | **o** | 0.16 | –0.05 | | **o** | 0.23 | –0.05 |
| | **m** | 0.17 | –0.04 | | **m** | 0.25 | –0.03 |
| | **p** | 0.20 | –0.01 | | **p** | 0.27 | –0.01 |
| $\sigma_{HOMO-5}$ | **B** | 0.07 | 0.00 | $\sigma_{HOMO-11}$ | **B** | 0.21 | 0.00 |
| | **o** | 0.09 | 0.02 | | **o** | 0.20 | –0.01 |
| | **m** | 0.05 | –0.02 | | **m** | 0.21 | 0.00 |
| | **p** | 0.16 | 0.09 | | **p** | 0.21 | 0.00 |
| $\sigma_{HOMO-6}$ | **B** | 0.18 | 0.00 | $\sigma_{HOMO-17}$ | **B** | 0.08 | 0.00 |
| | **o** | 0.12 | –0.06 | | **o** | 0.08 | 0.00 |
| | **m** | 0.19 | 0.01 | | **m** | 0.08 | 0.00 |
| | **p** | 0.09 | –0.09 | | **p** | 0.08 | 0.00 |

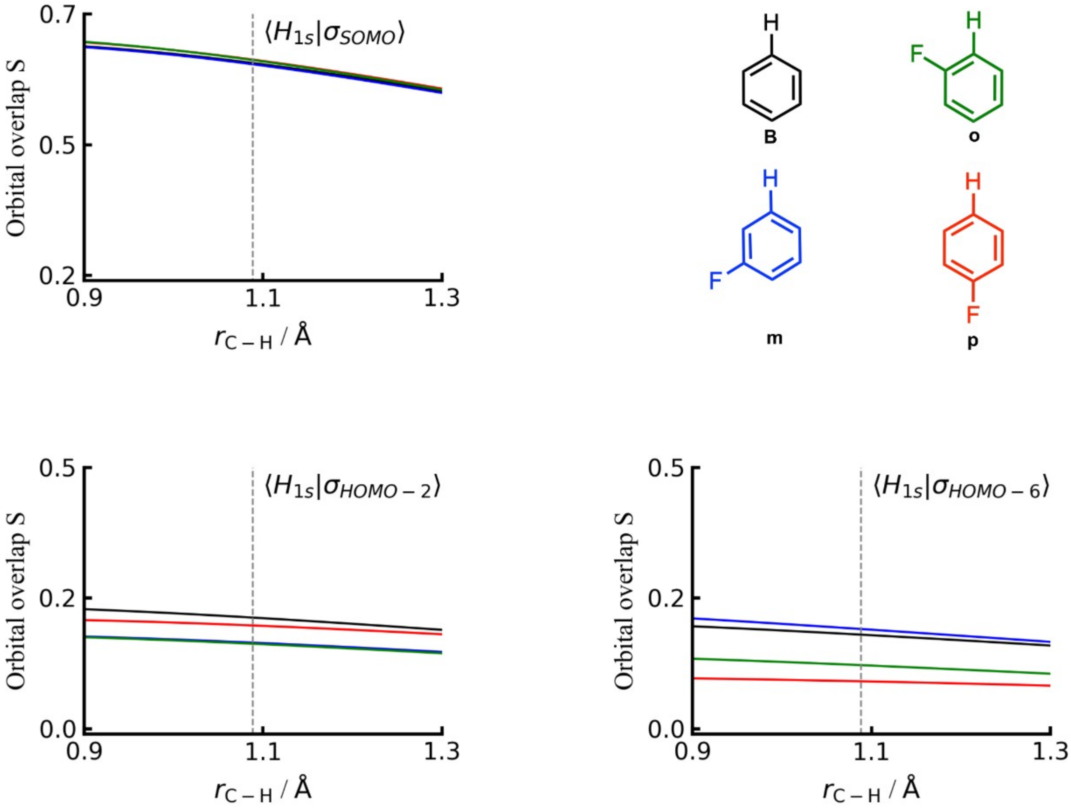


**Figure S9.** Orbital overlaps as a function of the C–H bond distance in benzene and fluorobenzene computed at ZORA-BLYP-D3(BJ)/TZ2P.

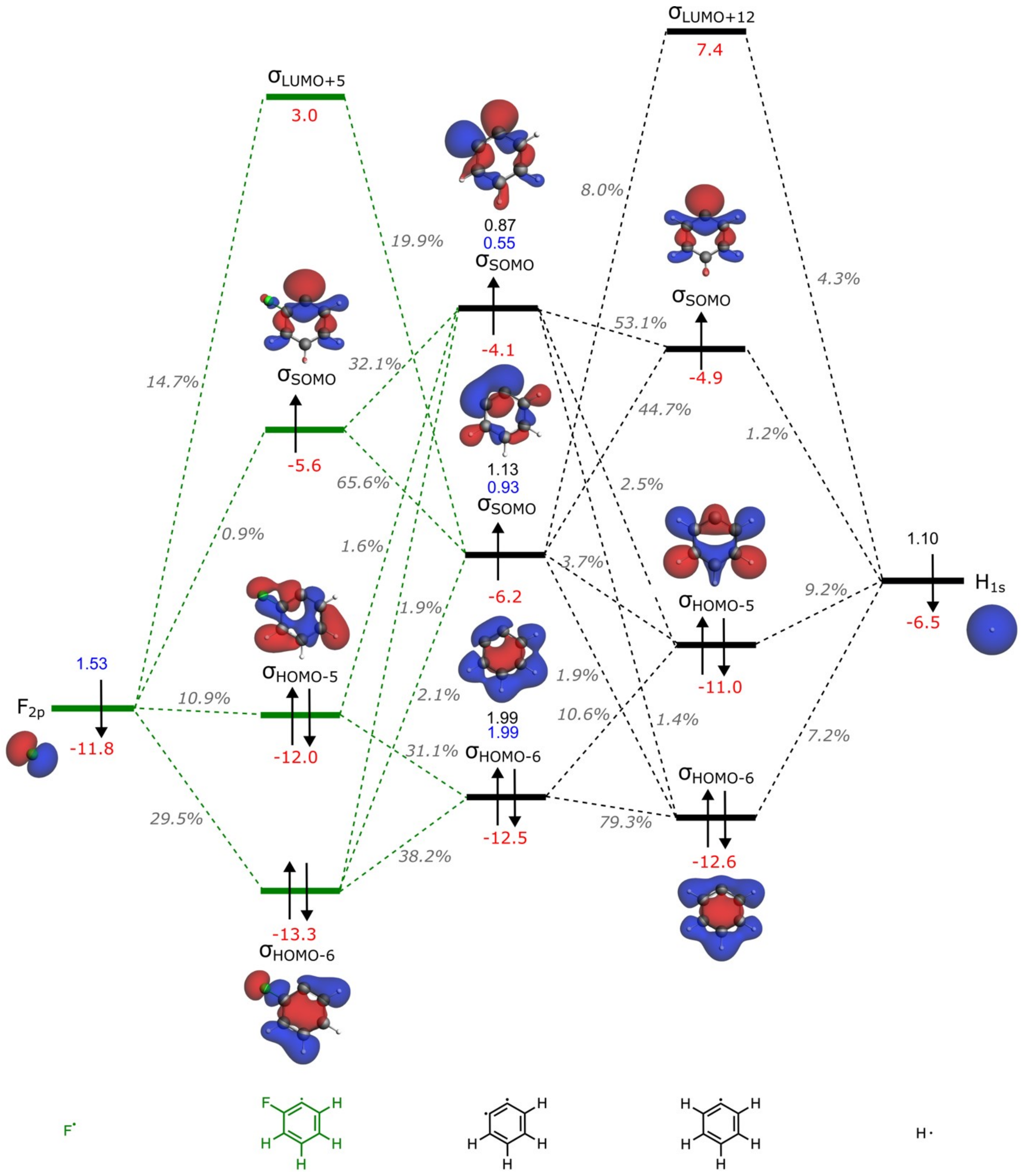


**Figure S10.** Qualitative MO diagram showing the formation of the $\sigma_{SOMO}$ and $\sigma_{HOMO\text{-}6}$ of the *ortho*-aryl radical ($C_6H_4R^{\bullet}$) from the interaction of $C_6H_4^{\bullet\bullet}$ + $R^{\bullet}$ (R = F *versus* H). The orbital energies (in eV) are given in red, gross population (in electrons) in blue (R = F) and black (R = H), and SFO Gross Mulliken contributions in gray. Computed at ZORA-BLYP-D3(BJ)/TZ2P.

**Table S4.** SFO Gross Mulliken contributions (%)[a] to the MOs of the *ortho*-aryl radical ($C_6H_4R^{\bullet}$) formed from the interaction of $C_6H_4^{\bullet\bullet}$ + $R^{\bullet}$ (R = F or H). Computed at ZORA-BLYP-D3(BJ)/TZ2P.

| MO | SFO Gross Mulliken contributions (%) | |
|---|---|---|
| | R = F | R = H |
| $\sigma_{SOMO}$ | 65.6% $\sigma_{HOMO-1}$<br>32.1% $\sigma_{HOMO}$ | 53.1% $\sigma_{HOMO}$<br>44.7% $\sigma_{HOMO-1}$<br>1.2% $H_{1s}$ |
| $\sigma_{HOMO-1}$ | 39.0% $\sigma_{HOMO-3}$<br>25.8% $\sigma_{HOMO-2}$<br>17.0% $F_{2px}$[b]<br>5.7% $\sigma_{HOMO-5}$<br>4.3% $\sigma_{HOMO-1}$<br>2.8% $F_{2pz}$[c]<br>2.3% $\sigma_{HOMO}$ | 48.6% $\sigma_{HOMO-2}$<br>19.0% $\sigma_{HOMO-1}$<br>10.8% $\sigma_{HOMO-3}$<br>10.0% $H_{1s}$<br>7.5% $\sigma_{HOMO}$<br>1.9% $\sigma_{HOMO-6}$<br>1.0% $\sigma_{HOMO-4}$ |
| $\sigma_{HOMO-2}$ | 49.9% $\sigma_{HOMO-3}$<br>22.6% $\sigma_{HOMO-2}$<br>14.5% $F_{2px}$[b]<br>10.0% $\sigma_{HOMO-5}$ | 51.5% $\sigma_{HOMO-3}$<br>38.6% $\sigma_{HOMO-2}$<br>3.2% $\sigma_{HOMO-1}$<br>3.2% $H_{1s}$<br>1.5% $\sigma_{HOMO}$ |
| $\sigma_{HOMO-3}$ | 89.3% $\sigma_{HOMO-4}$<br>2.8% $F_{2pz}$[c]<br>2.3% $\sigma_{HOMO-3}$<br>1.8% $\sigma_{HOMO-6}$<br>1.1% $\sigma_{HOMO-2}$ | 37.9% $\sigma_{HOMO-4}$<br>27.1% $\sigma_{HOMO-3}$<br>10.3% $H_{1s}$<br>7.8% $\sigma_{HOMO-2}$<br>5.5% $\sigma_{HOMO-6}$<br>5.4% $\sigma_{HOMO-1}$<br>3.3% $\sigma_{HOMO}$<br>1.5% $\sigma_{HOMO-7}$ |
| $\sigma_{HOMO-4}$ | 47.2% $\sigma_{HOMO-5}$<br>26.0% $\sigma_{HOMO-6}$<br>8.9% $F_{2px}$[b]<br>5.8% $F_{2pz}$[c]<br>3.0% $\sigma_{HOMO-4}$<br>2.9% $\sigma_{HOMO-2}$<br>2.2% $\sigma_{HOMO}$<br>1.0% $\sigma_{HOMO-8}$ | 99.5% $\sigma_{HOMO-5}$ |
| $\sigma_{HOMO-5}$ | 31.1% $\sigma_{HOMO-6}$<br>30.4% $\sigma_{HOMO-5}$<br>10.9% $F_{2pz}$[c]<br>7.1% $F_{2px}$[b]<br>5.1% $\sigma_{HOMO-7}$<br>5.0% $\sigma_{HOMO-4}$<br>4.1% $\sigma_{HOMO-3}$<br>2.2% $\sigma_{HOMO-1}$<br>1.7% $\sigma_{HOMO}$ | 59.4% $\sigma_{HOMO-4}$<br>10.6% $\sigma_{HOMO-6}$<br>9.2% $H_{1s}$<br>8.2% $\sigma_{HOMO-3}$<br>3.7% $\sigma_{HOMO-1}$<br>3.0% $\sigma_{HOMO-2}$<br>2.5% $\sigma_{HOMO}$<br>1.8% $\sigma_{HOMO-7}$ |
| $\sigma_{HOMO-6}$ | 38.2% $\sigma_{HOMO-6}$<br>29.5% $F_{2pz}$[c]<br>13.8% $\sigma_{HOMO-7}$<br>3.5% $\sigma_{HOMO-8}$<br>2.5% $F_{2s}$<br>2.1% $\sigma_{HOMO-1}$<br>1.9% $\sigma_{HOMO}$<br>1.6% $\sigma_{HOMO-9}$<br>1.5% $\sigma_{HOMO-3}$<br>1.1% $\sigma_{HOMO-4}$ | 79.3% $\sigma_{HOMO-6}$<br>7.2% $H_{1s}$<br>5.0% $\sigma_{HOMO-7}$<br>2.0% $\sigma_{HOMO-1}$<br>1.4% $\sigma_{HOMO}$<br>1.4% $\sigma_{HOMO-8}$<br>1.0% $\sigma_{HOMO-3}$ |

[a] Only contributions greater than 1% are listed. [b] doubly occupied orbital. [c] $F_{2pz}$ corresponds to the SOMO of the fluorine.

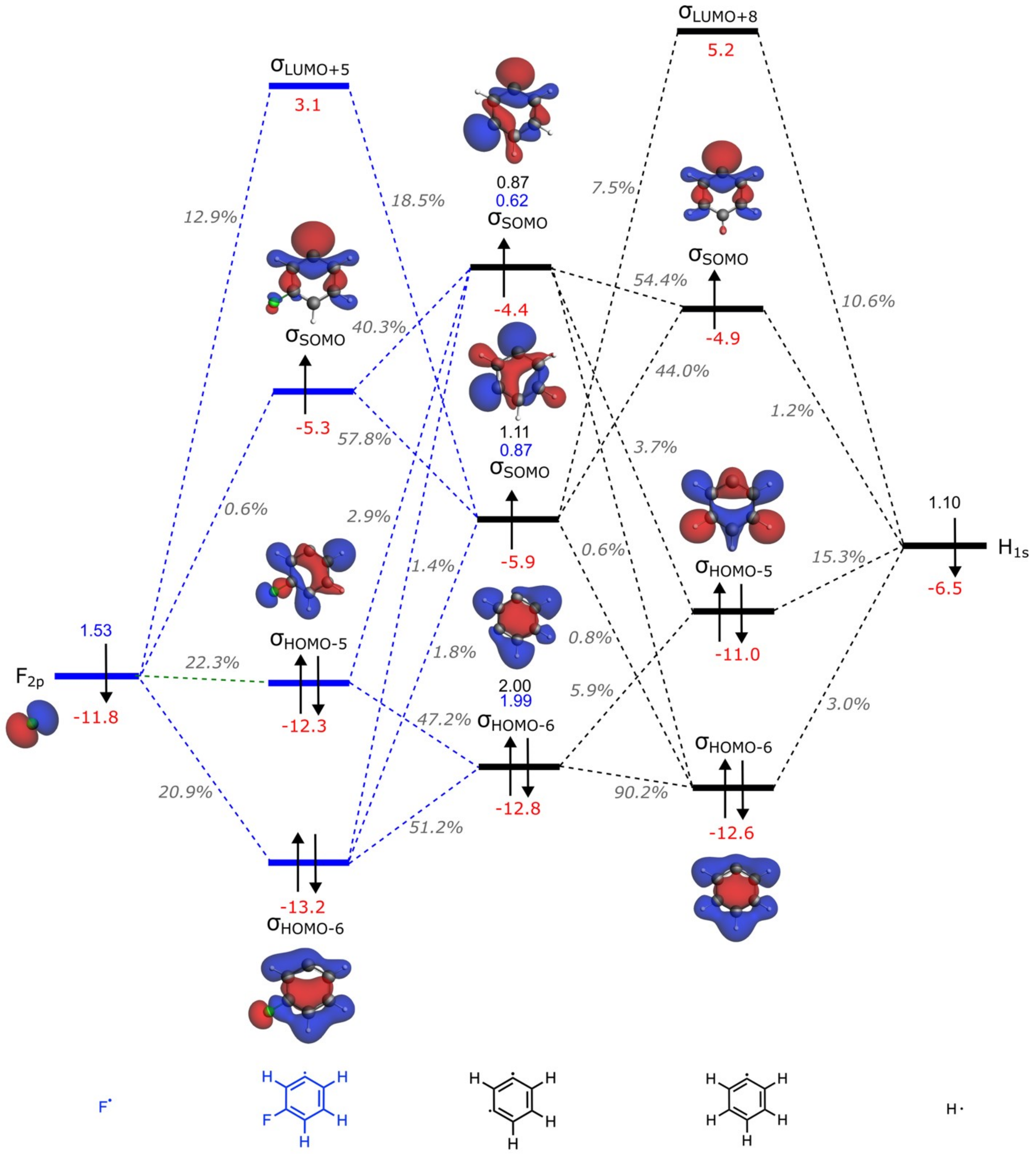


**Figure S11.** Qualitative MO diagram showing the formation of the $\sigma_{SOMO}$ and $\sigma_{HOMO-6}$ of the *meta*-aryl radical ($C_6H_4R^\bullet$) from the interaction of $C_6H_4^{\bullet\bullet}$ + $R^\bullet$ (R = F *versus* H). The orbital energies (in eV) are given in red, gross population (in electrons) in blue (R = F) and black (R = H), and SFO Gross Mulliken contributions in gray. Computed at ZORA-BLYP-D3(BJ)/TZ2P.

**Table S5.** SFO Gross Mulliken contributions (%)[a] for the MOs to the *meta*-aryl radical ($C_6H_4R^\bullet$) formed from the interaction of $C_6H_4^{\bullet\bullet}$ + $R^\bullet$ (R = F or H). Computed at ZORA-BLYP-D3(BJ)/TZ2P.

| MO | SFO Gross Mulliken contributions (%) | |
|---|---|---|
| | R = F | R = H |
| $\sigma_{SOMO}$ | 57.8% $\sigma_{HOMO-1}$<br>40.3% $\sigma_{HOMO}$ | 54.4% $\sigma_{HOMO}$<br>44.0% $\sigma_{HOMO-1}$<br>1.2% $H_{1s}$ |
| $\sigma_{HOMO-1}$ | 56.3% $\sigma_{HOMO-2}$<br>30.4% $F_{2px}$[b]<br>3.8% $\sigma_{HOMO-3}$<br>2.4% $\sigma_{HOMO}$<br>1.7% $\sigma_{HOMO-1}$<br>1.7% $F_{2pz}$[c]<br>1.3% $\sigma_{HOMO-4}$<br>1.0% $\sigma_{HOMO-3}$ | 49.8% $\sigma_{HOMO-2}$<br>18.3% $\sigma_{HOMO-1}$<br>10.8% $\sigma_{HOMO-3}$<br>9.9% $H_{1s}$<br>7.8% $\sigma_{HOMO}$<br>1.4% $\sigma_{HOMO-7}$ |
| $\sigma_{HOMO-2}$ | 67.8% $\sigma_{HOMO-3}$<br>16.4% $\sigma_{HOMO-2}$<br>7.8% $\sigma_{HOMO-5}$<br>7.0% $F_{2px}$[b] | 67.8% $\sigma_{HOMO-3}$<br>28.9% $\sigma_{HOMO-2}$<br>1.0% $\sigma_{HOMO-1}$ |
| $\sigma_{HOMO-3}$ | 92.9% $\sigma_{HOMO-4}$<br>1.6% $F_{2pz}$[c]<br>1.4% $\sigma_{HOMO-2}$<br>1.0% $\sigma_{HOMO-5}$ | 50.3% $\sigma_{HOMO-4}$<br>13.9% $\sigma_{HOMO-3}$<br>11.3% $\sigma_{HOMO-2}$<br>10.2% $H_{1s}$<br>5.7% $\sigma_{HOMO-1}$<br>3.2% $\sigma_{HOMO}$<br>2.3% $\sigma_{HOMO-7}$<br>2.0% $\sigma_{HOMO-6}$ |
| $\sigma_{HOMO-4}$ | 74.6% $\sigma_{HOMO-5}$<br>18.0% $F_{2px}$[b]<br>2.4% $\sigma_{HOMO-8}$<br>1.5% $\sigma_{HOMO-7}$ | 99.5% $\sigma_{HOMO-4}$<br>0.3% $H_{4s}$ |
| $\sigma_{HOMO-5}$ | 47.2% $\sigma_{HOMO-6}$<br>22.3% $F_{2pz}$[c]<br>10.0% $\sigma_{HOMO-7}$<br>4.0% $\sigma_{HOMO-1}$<br>3.9% $\sigma_{HOMO-4}$<br>3.7% $\sigma_{HOMO-2}$<br>2.9% $\sigma_{HOMO}$<br>1.2% $\sigma_{HOMO-3}$<br>1.0% $\sigma_{HOMO-9}$ | 47.7% $\sigma_{HOMO-5}$<br>15.3% $H_{1s}$<br>7.6% $\sigma_{HOMO-2}$<br>6.4% $\sigma_{HOMO-3}$<br>6.4% $\sigma_{HOMO-1}$<br>5.9% $\sigma_{HOMO-6}$<br>4.7% $\sigma_{HOMO-7}$<br>1.0% $\sigma_{HOMO-9}$ |
| $\sigma_{HOMO-6}$ | 51.2% $\sigma_{HOMO-6}$<br>20.9% $F_{2pz}$[c]<br>14.3% $\sigma_{HOMO-7}$<br>2.2% $\sigma_{HOMO-8}$<br>1.8% $\sigma_{HOMO-1}$<br>1.5% $F_{2s}$<br>1.5% $\sigma_{HOMO-2}$<br>1.4% $\sigma_{HOMO}$ | 90.2% $\sigma_{HOMO-6}$<br>3.5% $\sigma_{HOMO-7}$<br>3.0% $H_{1s}$ |

[a] Only contributions greater than 1% are listed. [b] doubly occupied orbital. [c] $F_{2pz}$ corresponds to the SOMO of the fluorine.

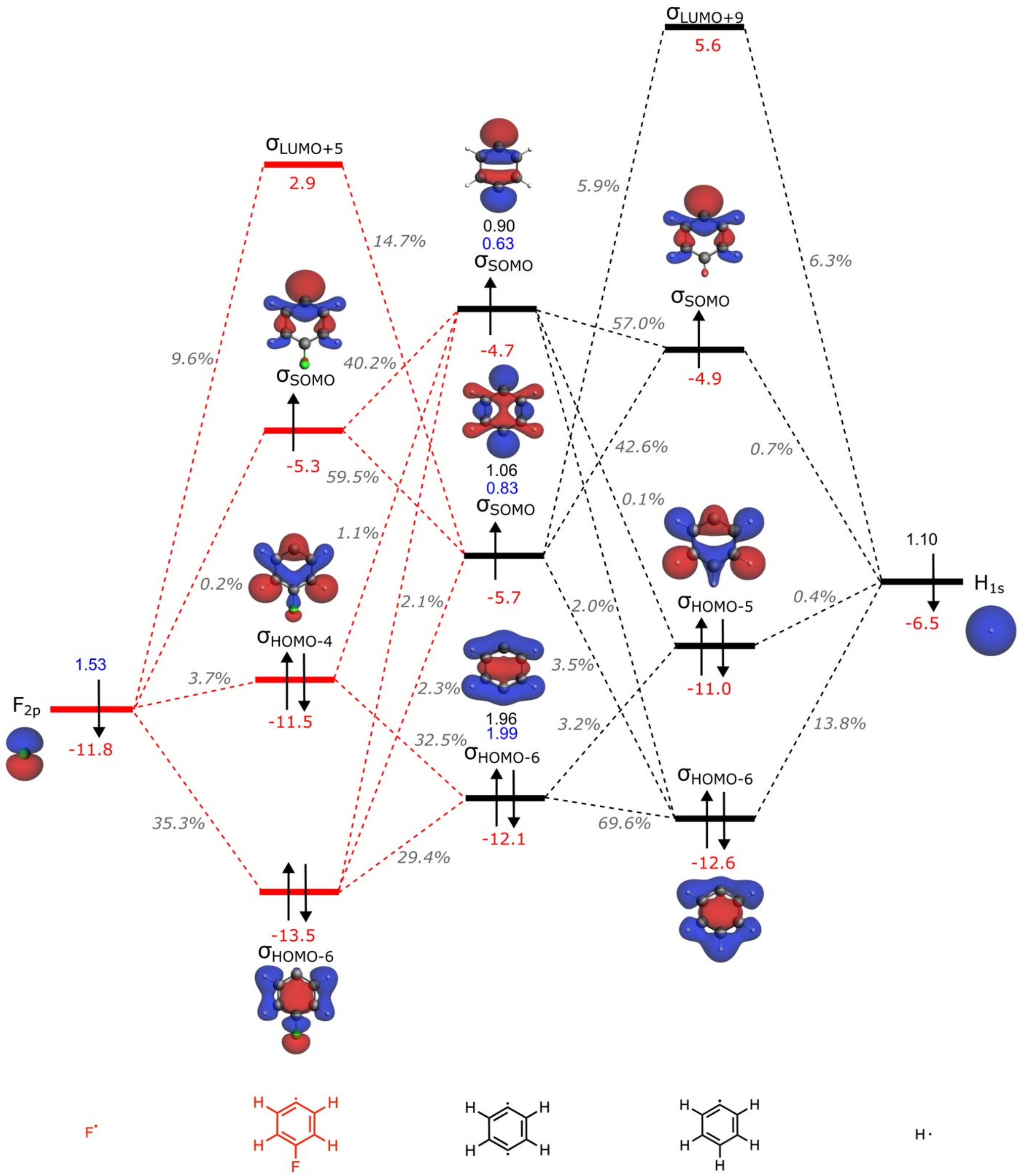


**Figure S12.** Qualitative MO diagram showing the formation of the $\sigma_{SOMO}$ and $\sigma_{HOMO\text{-}6}$ of the *para*-aryl radical ($C_6H_4R^{\bullet}$) from the interaction of $C_6H_4^{\bullet\bullet}$ + $R^{\bullet}$ (R = F *versus* H). The orbital energies (in eV) are given in red, gross population (in electrons) in blue (R = F) and black (R = H), and SFO Gross Mulliken contributions in gray. Computed at ZORA-BLYP-D3(BJ)/TZ2P.

**Table S6.** SFO Gross Mulliken contributions (%)[a] to the MOs of the *para*-aryl radical ($C_6H_4R^•$) formed from the interaction of $C_6H_4^{••}$ + $R^•$ (R = F or H). Computed at ZORA-BLYP-D3(BJ)/TZ2P.

| MO | SFO Gross Mulliken contributions (%) | |
|---|---|---|
| | R =F | R = H |
| $\sigma_{SOMO}$ | 59.5% $\sigma_{HOMO-1}$<br>40.2% $\sigma_{HOMO}$ | 57.0% $\sigma_{HOMO}$<br>42.6% $\sigma_{HOMO-1}$ |
| $\sigma_{HOMO-1}$ | 70.1% $\sigma_{HOMO-2}$<br>26.8% $F_{2px}$[b]<br>1.9% $\sigma_{HOMO-4}$ | 98.9% $\sigma_{HOMO-1}$ |
| $\sigma_{HOMO-2}$ | 37.5% $\sigma_{HOMO-5}$<br>33.7% $\sigma_{HOMO-6}$<br>14.3% $F_{2pz}$[c]<br>5.8% $\sigma_{HOMO-1}$<br>5.3% $\sigma_{HOMO}$<br>1.4% $\sigma_{HOMO-7}$ | 27.8% $H_{1s}$<br>27.3% $\sigma_{HOMO-2}$<br>23.4% $\sigma_{HOMO-6}$<br>13.9% $\sigma_{HOMO}$<br>3.0% $\sigma_{HOMO-5}$<br>2.6% $\sigma_{HOMO-7}$<br>2.2% $\sigma_{HOMO-9}$ |
| $\sigma_{HOMO-3}$ | 98.9% $\sigma_{HOMO-3}$ | 99.9% $\sigma_{HOMO-3}$ |
| $\sigma_{HOMO-4}$ | 76.1% $\sigma_{HOMO-4}$<br>18.4% $F_{2px}$[b]<br>3.1% $\sigma_{HOMO-8}$<br>1.8% $\sigma_{HOMO-2}$ | 99.4% $\sigma_{HOMO-4}$ |
| $\sigma_{HOMO-5}$ | 60.2% $\sigma_{HOMO-5}$<br>32.5% $\sigma_{HOMO-6}$<br>3.7% $F_{2pz}$[c]<br>1.1% $\sigma_{HOMO}$<br>1.1% $\sigma_{HOMO-1}$ | 95.9% $\sigma_{HOMO-5}$<br>3.2% $\sigma_{HOMO-6}$ |
| $\sigma_{HOMO-6}$ | 35.3% $F_{2pz}$[c]<br>29.4% $\sigma_{HOMO-6}$<br>20.2% $\sigma_{HOMO-7}$<br>2.9% $F_{2s}$<br>2.6% $\sigma_{HOMO-9}$<br>2.3% $\sigma_{HOMO-1}$<br>2.1% $\sigma_{HOMO}$<br>1.5% $\sigma_{HOMO-5}$ | 69.6% $\sigma_{HOMO-6}$<br>13.8% $H_{1s}$<br>7.3% $\sigma_{HOMO-7}$<br>3.5% $\sigma_{HOMO-1}$<br>2.0% $\sigma_{HOMO}$<br>2.0% $\sigma_{HOMO-9}$ |

[a] Only contributions greater than 1% are listed. [b] doubly occupied orbital. [c] $F_{2pz}$ corresponds to the SOMO of the fluorine.

**Table S7.** Energy decomposition analysis (in kcal mol$^{-1}$) of the *ortho*-C–H bond in *mono*-substituted benzenes $C_6H_5R$ (R= H, F, Cl, Br, I, and Li) at a consistent geometry[a] with a C–H distance of 1.088 Å. Computed at ZORA-BLYP-D3(BJ)/TZ2P.

| R | $\Delta H$ | $\Delta E$ | $\Delta E_{strain}$ | $\Delta E_{int}$ | $\Delta V_{elstat}$ | $\Delta E_{Pauli}$ | $\Delta E_{oi}$ | $\Delta E_{pb}$ | $\Delta E_{disp}$ | $\Delta E_{spinpol}$ |
|---|---|---|---|---|---|---|---|---|---|---|
| **H** | –109.3 | –115.9 | 1.8 | –117.7 | –64.9 | 94.9 | –149.5 | –96.5 | –0.9 | 2.7 |
| **F** | –111.8 | –118.4 | 1.8 | –120.2 | –61.9 | 90.1 | –150.2 | –96.6 | –1.0 | 2.8 |
| **Cl** | –110.9 | –117.5 | 1.9 | –119.5 | –63.0 | 93.0 | –151.1 | –98.4 | –1.1 | 2.8 |
| **Br** | –110.3 | –117.0 | 2.0 | –119.0 | –63.2 | 95.8 | –153.3 | –100.2 | –1.2 | 2.8 |
| **I** | –109.4 | –116.0 | 2.1 | –118.1 | –64.6 | 101.6 | –156.8 | –102.9 | –1.2 | 3.0 |
| **Li** | –91.9 | –98.1 | 10.7 | –108.9 | –74.6 | 125.3 | –161.1 | –99.0 | –1.0 | 2.6 |

[a] The equilibrium C–H bond length in benzene.

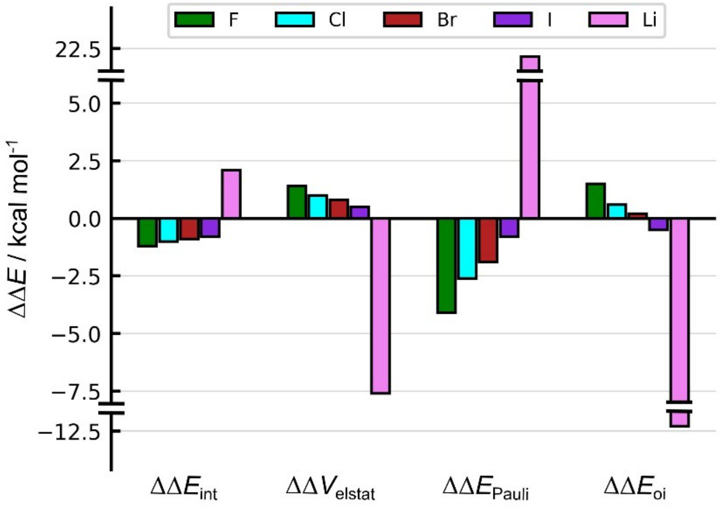


**Figure S13.** Energy decomposition analysis of the *para*-C–H bond in mono-substituted benzenes $C_6RH_5$ (R = F, Cl, Br, I, and Li) relative to benzene at a consistent geometry with a C–H distance of 1.088 Å. Computed at ZORA-BLYP-D3(BJ)/TZ2P.

**Table S8**. Cartesian coordinates (in Å), energies (electronic energy E and enthalpy H, in kcal mol$^{-1}$), number of imaginary frequencies ($N_{imag}$), and multiplicity (2S + 1) of all systems studied herein, computed at ZORA-BLYP-D3(BJ)/TZ2P.

**$C_6H_6$ (B)**
**E** = -1680.98
**H** = -1616.32
***$N_{imag}$*** = 0
**2S+1** = 1

| | | | |
|---|---|---|---|
| C | -0.699921 | 1.212266 | 0.000000 |
| C | 0.000019 | -0.000000 | 0.000000 |
| C | -0.699921 | -1.212266 | 0.000000 |
| C | -2.099744 | -1.212266 | 0.000000 |
| C | -2.799685 | -0.000000 | 0.000000 |
| C | -2.099744 | 1.212266 | 0.000000 |
| H | 1.088024 | -0.000000 | 0.000000 |
| H | -0.155941 | -2.154521 | 0.000000 |
| H | -2.643725 | -2.154521 | 0.000000 |
| H | -3.887690 | -0.000000 | 0.000000 |
| H | -2.643725 | 2.154521 | 0.000000 |
| H | -0.155941 | 2.154521 | 0.000000 |

**$C_6FH_5$**
***E*** = -1686.67
***H*** = -1626.62
***$N_{imag}$*** = 0
**2S+1** = 1

| | | | |
|---|---|---|---|
| C | -0.705920 | -1.211576 | 0.000000 |
| C | -0.004809 | -0.000002 | 0.000000 |
| C | -0.705934 | 1.211563 | 0.000000 |
| C | -2.105681 | 1.221204 | 0.000000 |
| C | -2.773576 | -0.000017 | 0.000000 |
| C | -2.105667 | -1.221232 | -0.000000 |
| H | 1.082206 | 0.000007 | 0.000000 |
| H | -0.165590 | 2.155299 | 0.000000 |
| H | -2.672926 | 2.147805 | 0.000000 |
| H | -2.672901 | -2.147839 | -0.000000 |
| H | -0.165564 | -2.155304 | -0.000000 |
| F | -4.146735 | -0.000025 | 0.000000 |

**1,2-$C_6F_2H_4$**
***E*** = -1687.79
***H*** = -1632.25
***$N_{imag}$*** = 0
**2S + 1** = 1

| | | | |
|---|---|---|---|
| C | -0.696861 | 1.222249 | 0.000000 |
| C | -2.087865 | 1.220555 | 0.000000 |
| C | -2.796240 | 0.015676 | 0.000000 |
| C | -2.121220 | -1.200588 | 0.000000 |
| C | -0.721141 | -1.204952 | 0.000000 |
| C | -0.012134 | 0.001029 | 0.000000 |
| F | -2.775566 | 2.397130 | 0.000000 |
| F | -4.158751 | 0.044449 | 0.000000 |
| H | -2.697448 | -2.121902 | 0.000000 |

| | | | |
|---|---|---|---|
| H | -0.189222 | -2.152495 | 0.000000 |
| H | -0.172049 | 2.173797 | 0.000000 |
| H | 1.074490 | -0.002972 | 0.000000 |

**1,3-$C_6F_2H_4$**
***E*** = -1691.54
***H*** = -1636.1
$N_{imag}$ = 0
**2S+1** = 1

| | | | |
|---|---|---|---|
| C | -0.714469 | 1.186415 | 0.000000 |
| C | 0.015198 | -0.000000 | 0.000000 |
| C | -0.714469 | -1.186415 | 0.000000 |
| C | -2.106607 | -1.218461 | 0.000000 |
| C | -2.795095 | -0.000000 | 0.000000 |
| C | -2.106607 | 1.218461 | 0.000000 |
| H | 1.100322 | -0.000000 | 0.000000 |
| F | -0.024542 | -2.369034 | 0.000000 |
| H | -2.626624 | -2.171406 | 0.000000 |
| H | -3.881956 | -0.000000 | 0.000000 |
| H | -2.626624 | 2.171406 | 0.000000 |
| F | -0.024542 | 2.369034 | 0.000000 |

**1,4-$C_6F_2H_4$**
***E*** = -1690.93
***H*** = -1635.52
$N_{imag}$ = 0
**2S + 1** = 1

| | | | |
|---|---|---|---|
| C | 0.000000 | 1.220583 | 0.699724 |
| C | 0.000000 | 0.000000 | 1.369737 |
| C | -0.000000 | -1.220583 | 0.699724 |
| C | -0.000000 | -1.220583 | -0.699724 |
| C | -0.000000 | 0.000000 | -1.369737 |
| C | 0.000000 | 1.220583 | -0.699724 |
| F | 0.000000 | 0.000000 | 2.741728 |
| H | 0.000000 | -2.148780 | 1.263656 |
| H | 0.000000 | -2.148780 | -1.263656 |
| F | -0.000000 | 0.000000 | -2.741728 |
| H | -0.000000 | 2.148780 | 1.263656 |
| H | -0.000000 | 2.148780 | -1.263656 |

**1,3,5-$C_6F_3H_3$**
***E*** = -1695.53
***H*** = -1644.7
$N_{imag}$ = 0
**2S+1** = 1

| | | | |
|---|---|---|---|
| C | -0.715204 | 1.185826 | 0.000000 |
| C | 0.015851 | -0.000000 | 0.000000 |
| C | -0.715204 | -1.185826 | 0.000000 |
| C | -2.107689 | -1.226046 | 0.000000 |
| C | -2.769133 | -0.000000 | 0.000000 |
| C | -2.107689 | 1.226046 | 0.000000 |
| H | 1.100327 | -0.000000 | 0.000000 |
| F | -0.032443 | -2.368300 | 0.000000 |
| H | -2.649899 | -2.165247 | 0.000000 |

| | | | |
|---|---|---|---|
| F | -4.134578 | -0.000000 | 0.000000 |
| H | -2.649899 | 2.165247 | 0.000000 |
| F | -0.032443 | 2.368300 | 0.000000 |

**1,2,3-$C_6F_3H_3$**
***E*** = -1688.34
***H*** = -1637.27
$N_{imag}$ = 0
**2S+1** = 1

| | | | |
|---|---|---|---|
| C | -0.696285 | 1.216849 | 0.000000 |
| C | -0.005971 | 0.000000 | 0.000000 |
| C | -0.696285 | -1.216849 | 0.000000 |
| C | -2.087933 | -1.202596 | 0.000000 |
| C | -2.800527 | -0.000000 | 0.000000 |
| C | -2.087933 | 1.202596 | 0.000000 |
| H | 1.080158 | 0.000000 | 0.000000 |
| H | -0.178149 | -2.171175 | 0.000000 |
| F | -2.788389 | -2.368960 | 0.000000 |
| F | -4.154131 | -0.000000 | 0.000000 |
| F | -2.788390 | 2.368960 | 0.000000 |
| H | -0.178149 | 2.171175 | 0.000000 |

**1,2,4-$C_6F_3H_3$**
***E*** = -1691.47
***H*** = -1640.55
$N_{imag}$ = 0
**2S + 1** = 1

| | | | |
|---|---|---|---|
| C | -0.701434 | 1.197587 | 0.000000 |
| C | 0.001883 | -0.002258 | 0.000000 |
| C | -0.693970 | -1.217131 | 0.000000 |
| C | -2.085302 | -1.186899 | 0.000000 |
| C | -2.812850 | 0.001768 | 0.000000 |
| C | -2.099802 | 1.197052 | 0.000000 |
| H | 1.087786 | 0.023501 | 0.000000 |
| H | -0.170119 | -2.167698 | 0.000000 |
| F | -2.775539 | -2.368653 | 0.000000 |
| H | -3.898214 | 0.003246 | 0.000000 |
| F | -2.767810 | 2.380854 | 0.000000 |
| F | -0.035422 | 2.385818 | 0.000000 |

**1,2,3,4-$C_6F_4H_2$**
***E*** = -1687.83
***H*** = -1641.26
$N_{imag}$ = 0
**2S + 1** = 1

| | | | |
|---|---|---|---|
| C | -0.703251 | 1.197175 | 0.000000 |
| C | 0.000786 | -0.001626 | 0.000000 |
| C | -0.699200 | -1.213939 | 0.000000 |
| C | -2.089402 | -1.203595 | 0.000000 |
| C | -2.800890 | 0.000642 | 0.000000 |
| C | -2.101887 | 1.211258 | 0.000000 |
| H | 1.086093 | 0.021690 | 0.000000 |
| H | -0.176773 | -2.165526 | 0.000000 |

| | | | |
|---|---|---|---|
| F | -2.789366 | -2.369876 | 0.000000 |
| F | -4.152405 | 0.004894 | 0.000000 |
| F | -2.781296 | 2.379621 | 0.000000 |
| F | -0.043203 | 2.386473 | 0.000000 |

**1,2,4,5-$C_6F_4H_2$**
***E*** = -1690.93
***H*** = -1644.52
$N_{imag}$ = 0
**2S+1** = 1

| | | | |
|---|---|---|---|
| C | -0.701087 | 1.198327 | 0.000000 |
| C | 0.008042 | -0.000000 | 0.000000 |
| C | -0.701087 | -1.198327 | 0.000000 |
| C | -2.098580 | -1.198327 | 0.000000 |
| C | -2.807709 | -0.000000 | 0.000000 |
| C | -2.098580 | 1.198327 | 0.000000 |
| H | 1.093592 | 0.000000 | 0.000000 |
| F | -0.030916 | -2.380713 | 0.000000 |
| F | -2.768751 | -2.380713 | 0.000000 |
| H | -3.893259 | -0.000000 | 0.000000 |
| F | -2.768751 | 2.380713 | 0.000000 |
| F | -0.030916 | 2.380713 | 0.000000 |

**1,2,3,5-$C_6F_4H_2$**
***E*** = -1691.23
***H*** = -1644.79
$N_{imag}$ = 0
**2S + 1** = 1

| | | | |
|---|---|---|---|
| C | -0.716229 | 1.183392 | 0.000000 |
| C | 0.012439 | -0.003310 | 0.000000 |
| C | -0.703474 | -1.197870 | 0.000000 |
| C | -2.101318 | -1.215667 | 0.000000 |
| C | -2.784863 | 0.003810 | 0.000000 |
| C | -2.108277 | 1.221104 | 0.000000 |
| H | 1.097255 | -0.005677 | 0.000000 |
| F | -0.040172 | -2.381877 | 0.000000 |
| F | -2.778037 | -2.387737 | 0.000000 |
| F | -4.141896 | -0.013836 | 0.000000 |
| H | -2.652713 | 2.159416 | 0.000000 |
| F | -0.033507 | 2.365825 | 0.000000 |

**1,2,3,4,5-$C_6F_5H$**
***E*** = -1686.59
***H*** = -1644.52
$N_{imag}$ = 0
**2S+1** = 1

| | | | |
|---|---|---|---|
| C | -0.703448 | 1.196711 | 0.000000 |
| C | 0.007611 | -0.000000 | 0.000000 |
| C | -0.703448 | -1.196711 | 0.000000 |
| C | -2.100596 | -1.213119 | 0.000000 |
| C | -2.796925 | -0.000000 | 0.000000 |
| C | -2.100596 | 1.213119 | 0.000000 |
| H | 1.092728 | 0.000000 | 0.000000 |

| | | | |
|---|---|---|---|
| F | -0.037856 | -2.379615 | 0.000000 |
| F | -2.781569 | -2.380623 | 0.000000 |
| F | -4.146470 | -0.000000 | 0.000000 |
| F | -2.781569 | 2.380623 | 0.000000 |
| F | -0.037856 | 2.379615 | 0.000000 |

**$C_6ClH_5$**
***E*** = -1644.02
***H*** = -1584.52
$N_{imag}$ = 0
**2S+1** = 1

| | | | |
|---|---|---|---|
| C | -0.000000 | 1.210060 | 0.755698 |
| C | 0.000000 | -0.000000 | 1.458037 |
| C | 0.000000 | -1.210060 | 0.755698 |
| C | 0.000000 | -1.218540 | -0.643299 |
| C | 0.000000 | 0.000000 | -1.326354 |
| C | -0.000000 | 1.218540 | -0.643299 |
| H | 0.000000 | -0.000000 | 2.545199 |
| H | 0.000000 | -2.155285 | 1.293828 |
| H | -0.000000 | -2.152559 | -1.197188 |
| H | 0.000000 | 2.152559 | -1.197188 |
| H | -0.000000 | 2.155285 | 1.293828 |
| Cl | 0.000000 | -0.000000 | -3.094961 |

**$C_6BrH_5$**
***E*** = -1630.83
***H*** = -1571.52
$N_{imag}$ = 0
**2S+1** = 1

| | | | |
|---|---|---|---|
| C | -0.000000 | 1.210195 | 0.754897 |
| C | 0.000000 | 0.000000 | 1.456762 |
| C | 0.000000 | -1.210195 | 0.754897 |
| C | 0.000000 | -1.219194 | -0.644925 |
| C | 0.000000 | -0.000000 | -1.324539 |
| C | -0.000000 | 1.219194 | -0.644925 |
| H | 0.000000 | 0.000000 | 2.543941 |
| H | -0.000000 | -2.155628 | 1.292792 |
| H | -0.000000 | -2.155029 | -1.195293 |
| H | 0.000000 | 2.155029 | -1.195293 |
| H | 0.000000 | 2.155628 | 1.292792 |
| Br | 0.000000 | 0.000000 | -3.261108 |

**$C_6IH_5$**
***E*** = -1617.95
***H*** = -1558.76
$N_{imag}$ = 0
**2S+1** = 1

| | | | |
|---|---|---|---|
| C | -0.000000 | 1.209810 | 0.755188 |
| C | 0.000000 | 0.000000 | 1.457273 |
| C | 0.000000 | -1.209810 | 0.755188 |
| C | 0.000000 | -1.218423 | -0.644923 |
| C | 0.000000 | -0.000000 | -1.329964 |
| C | -0.000000 | 1.218423 | -0.644923 |
| H | 0.000000 | 0.000000 | 2.544526 |
| H | -0.000000 | -2.155659 | 1.292674 |

| | | | |
|---|---|---|---|
| H | -0.000000 | -2.158276 | -1.188519 |
| H | 0.000000 | 2.158276 | -1.188519 |
| H | 0.000000 | 2.155659 | 1.292674 |
| I | 0.000000 | 0.000000 | -3.470675 |

**$C_6LiH_5$**
***E*** = -1605.15
***H*** = -1546.55
$N_{imag}$ = 0
**2S+1** = 1

| | | | |
|---|---|---|---|
| C | -0.000000 | 1.206409 | 0.734846 |
| C | 0.000000 | -0.000000 | 1.444272 |
| C | 0.000000 | -1.206409 | 0.734846 |
| C | 0.000000 | -1.193869 | -0.668196 |
| C | 0.000000 | 0.000000 | -1.435750 |
| C | -0.000000 | 1.193869 | -0.668196 |
| H | 0.000000 | -0.000000 | 2.533123 |
| H | 0.000000 | -2.153910 | 1.274768 |
| H | -0.000000 | -2.164958 | -1.172433 |
| H | 0.000000 | 2.164958 | -1.172433 |
| H | -0.000000 | 2.153910 | 1.274768 |
| Li | 0.000000 | -0.000000 | -3.399617 |

**$C_6H_5^{\cdot}$ ($B^{\cdot}$)**
***E*** = -1542.88
***H*** = -1486.38
$N_{imag}$ = 0
**2S+1** = 2

| | | | |
|---|---|---|---|
| C | -0.689910 | 1.227330 | 0.000000 |
| C | -0.064741 | -0.000000 | 0.000000 |
| C | -0.689910 | -1.227330 | 0.000000 |
| C | -2.099806 | -1.216435 | 0.000000 |
| C | -2.792998 | -0.000000 | 0.000000 |
| C | -2.099806 | 1.216435 | 0.000000 |
| H | -0.140402 | 2.166603 | 0.000000 |
| H | -0.140402 | -2.166603 | 0.000000 |
| H | -2.643725 | -2.159696 | 0.000000 |
| H | -3.880579 | -0.000000 | 0.000000 |
| H | -2.643725 | 2.159696 | 0.000000 |

**2-$C_6FH_4^{\cdot}$ (o)**
***E*** = -1546.14
***H*** = -1494.12
$N_{imag}$ = 0
**2S+1** = 2

| | | | |
|---|---|---|---|
| C | -0.705767 | 1.211096 | 0.000000 |
| C | -0.055702 | -0.000786 | 0.000000 |
| C | -0.687207 | -1.221261 | 0.000000 |
| C | -2.098460 | -1.214868 | 0.000000 |
| C | -2.790741 | 0.001287 | 0.000000 |
| C | -2.104652 | 1.222570 | 0.000000 |
| F | -0.017255 | 2.392556 | 0.000000 |
| H | -0.134615 | -2.158913 | 0.000000 |
| H | -2.638726 | -2.158861 | 0.000000 |
| H | -3.877830 | 0.003948 | 0.000000 |

| | | | |
|---|---|---|---|
| H | -2.629055 | 2.176102 | 0.000000 |

**3-$C_6FH_4^•$ (m)**
***E*** = -1548.2
***H*** = -1496.28
$N_{imag}$ = 0
**2S+1** = 2

| | | | |
|---|---|---|---|
| C | -0.684033 | 1.233753 | 0.000000 |
| C | -0.068485 | 0.001910 | 0.000000 |
| C | -0.689185 | -1.227588 | 0.000000 |
| C | -2.098849 | -1.212767 | 0.000000 |
| C | -2.801332 | -0.002559 | 0.000000 |
| C | -2.085262 | 1.193571 | 0.000000 |
| H | -0.155770 | 2.184183 | 0.000000 |
| H | -0.140013 | -2.165813 | 0.000000 |
| H | -2.646118 | -2.153705 | 0.000000 |
| H | -3.886943 | 0.024140 | 0.000000 |
| F | -2.776009 | 2.377744 | 0.000000 |

**4-$C_6FH_4^•$ (p)**
***E*** = -1547.3
***H*** = -1495.38
$N_{imag}$ = 0
**2S+1** = 2

| | | | |
|---|---|---|---|
| C | -0.691958 | 1.226989 | 0.000000 |
| C | -0.064816 | -0.000000 | 0.000000 |
| C | -0.691958 | -1.226989 | 0.000000 |
| C | -2.100770 | -1.225952 | 0.000000 |
| C | -2.763665 | -0.000000 | 0.000000 |
| C | -2.100770 | 1.225952 | 0.000000 |
| H | -0.143959 | 2.166615 | 0.000000 |
| H | -0.143959 | -2.166615 | 0.000000 |
| H | -2.669716 | -2.152315 | 0.000000 |
| F | -4.136706 | -0.000000 | 0.000000 |
| H | -2.669716 | 2.152315 | 0.000000 |

**2,3-$C_6F_2H_3^•$ (o-m)**
***E*** = -1547.32
***H*** = -1499.73
$N_{imag}$ = 0
**2S + 1** = 2

| | | | |
|---|---|---|---|
| C | -0.671576 | 1.238402 | 0.000000 |
| C | -2.076089 | 1.257212 | 0.000000 |
| C | -2.806328 | 0.069970 | 0.000000 |
| C | -2.136656 | -1.158403 | 0.000000 |
| C | -0.727181 | -1.205499 | 0.000000 |
| C | -0.072787 | 0.002992 | 0.000000 |
| F | -2.720726 | 2.457524 | 0.000000 |
| H | -3.891102 | 0.124460 | 0.000000 |
| H | -2.704821 | -2.085740 | 0.000000 |
| H | -0.197999 | -2.155374 | 0.000000 |
| F | 0.030047 | 2.402356 | 0.000000 |

**3,4-$C_6F_2H_3^{\cdot}$ (m-p)**
***E*** = -1548.32
***H*** = -1500.88
$N_{imag}$ = 0
**2S + 1** = 2

| | | | |
|---|---|---|---|
| C | -0.666152 | 1.249336 | 0.000000 |
| C | -2.066096 | 1.263415 | 0.000000 |
| C | -2.789145 | 0.063953 | 0.000000 |
| C | -2.141380 | -1.167996 | 0.000000 |
| C | -0.732720 | -1.209623 | 0.000000 |
| C | -0.079533 | 0.002364 | 0.000000 |
| F | -2.739934 | 2.445497 | 0.000000 |
| F | -4.151013 | 0.117344 | 0.000000 |
| H | -2.735603 | -2.078847 | 0.000000 |
| H | -0.209612 | -2.162401 | 0.000000 |
| H | -0.113351 | 2.185892 | 0.000000 |

**2,6-$C_6F_2H_3^{\cdot}$ (2o)**
***E*** = -1548.25
***H*** = -1500.68
$N_{imag}$ = 0
**2S+1** = 2

| | | | |
|---|---|---|---|
| C | -0.703042 | 1.208413 | 0.000000 |
| C | -0.047226 | -0.000000 | 0.000000 |
| C | -0.703042 | -1.208413 | 0.000000 |
| C | -2.103242 | -1.221420 | 0.000000 |
| C | -2.787110 | -0.000000 | 0.000000 |
| C | -2.103242 | 1.221420 | 0.000000 |
| F | -0.011377 | 2.383909 | 0.000000 |
| F | -0.011377 | -2.383909 | 0.000000 |
| H | -2.625348 | -2.175186 | 0.000000 |
| H | -3.873660 | -0.000000 | 0.000000 |
| H | -2.625348 | 2.175186 | 0.000000 |

**3,5-$C_6F_2H_3^{\cdot}$ (2m)**
***E*** = -1552.59
***H*** = -1505.25
$N_{imag}$ = 0
**2S+1** = 2

| | | | |
|---|---|---|---|
| C | -2.080103 | 1.192403 | 0.000000 |
| C | -2.802453 | 0.000000 | 0.000000 |
| C | -2.080103 | -1.192403 | 0.000000 |
| C | -0.678443 | -1.234388 | 0.000000 |
| C | -0.069492 | 0.000000 | 0.000000 |
| C | -0.678443 | 1.234388 | 0.000000 |
| H | -3.887123 | 0.000000 | 0.000000 |
| F | -2.774174 | -2.370870 | 0.000000 |
| H | -0.148737 | -2.183068 | 0.000000 |
| F | -2.774174 | 2.370870 | 0.000000 |
| H | -0.148737 | 2.183068 | 0.000000 |

**2,4-$C_6F_2H_3^{\cdot}$ (o-p)**
***E*** = -1549.88
***H*** = -1502.43

$N_{imag}$ = 0
**2S + 1** = 2

| | | | |
|---|---|---|---|
| C | -0.684775 | 1.228080 | 0.000000 |
| C | -2.082322 | 1.283078 | 0.000000 |
| C | -2.771535 | 0.069665 | 0.000000 |
| C | -2.139824 | -1.172454 | 0.000000 |
| C | -0.730379 | -1.202975 | 0.000000 |
| C | -0.064554 | -0.000433 | 0.000000 |
| H | -2.608566 | 2.233776 | 0.000000 |
| F | -4.139910 | 0.109462 | 0.000000 |
| H | -2.727463 | -2.085879 | 0.000000 |
| H | -0.203419 | -2.154759 | 0.000000 |
| F | 0.029743 | 2.389761 | 0.000000 |

**2,5-$C_6F_2H_3^{\cdot}$ (o-m')**
***E*** = -1550.08
***H*** = -1502.65
$N_{imag}$ = 0
**2S + 1** = 2

| | | | |
|---|---|---|---|
| C | -0.057530 | -0.001771 | 0.000000 |
| C | -0.705168 | 1.211522 | 0.000000 |
| C | -2.103970 | 1.217284 | 0.000000 |
| C | -2.798542 | 0.001518 | 0.000000 |
| C | -2.082185 | -1.193775 | 0.000000 |
| C | -0.678939 | -1.227354 | 0.000000 |
| F | -0.018476 | 2.392643 | 0.000000 |
| H | -2.633000 | 2.168071 | 0.000000 |
| H | -3.883878 | -0.023228 | 0.000000 |
| F | -2.766354 | -2.379604 | 0.000000 |
| H | -0.147308 | -2.176132 | 0.000000 |

**2,3,4-$C_6F_3H_2^{\cdot}$ (o-m-p)**
***E*** = -1546.98
***H*** = -1503.89
$N_{imag}$ = 0
**2S + 1** = 2

| | | | |
|---|---|---|---|
| C | -0.069154 | 0.000192 | 0.000000 |
| C | -0.729768 | -1.204141 | 0.000000 |
| C | -2.138714 | -1.165434 | 0.000000 |
| C | -2.782536 | 0.068756 | 0.000000 |
| C | -2.069787 | 1.275380 | 0.000000 |
| C | -0.666782 | 1.237340 | 0.000000 |
| H | -0.204932 | -2.156215 | 0.000000 |
| H | -2.730852 | -2.076623 | 0.000000 |
| F | -4.141677 | 0.128774 | 0.000000 |
| F | -2.726389 | 2.458672 | 0.000000 |
| F | 0.034939 | 2.398709 | 0.000000 |

**2,3,6-$C_6F_3H_2^{\cdot}$ (2o-m)**
***E*** = -1548.25
***H*** = -1505.15
$N_{imag}$ = 0

**2S + 1** = 2

| | | | |
|---|---|---|---|
| C | -2.062034 | 1.203554 | 0.000000 |
| C | -2.759859 | -0.001403 | 0.000000 |
| C | -2.066552 | -1.217912 | 0.000000 |
| C | -0.667798 | -1.209782 | 0.000000 |
| C | -0.017917 | 0.002267 | 0.000000 |
| C | -0.655588 | 1.218087 | 0.000000 |
| H | -3.845383 | 0.021922 | 0.000000 |
| H | -2.593410 | -2.169140 | 0.000000 |
| F | 0.024448 | -2.383890 | 0.000000 |
| F | 0.017263 | 2.395027 | 0.000000 |
| F | -2.731972 | 2.388458 | 0.000000 |

**2,3,5-$C_6F_3H_2^{\bullet}$ (o-2m)**
***E*** = -1550.48
***H*** = -1507.50
***Nimag*** = 0
**2S + 1** = 2

| | | | |
|---|---|---|---|
| C | -0.693898 | 1.222568 | 0.000000 |
| C | -0.069798 | 0.000244 | 0.000000 |
| C | -0.683172 | -1.229350 | 0.000000 |
| C | -2.085455 | -1.191508 | 0.000000 |
| C | -2.805980 | 0.001848 | 0.000000 |
| C | -2.098728 | 1.203216 | 0.000000 |
| H | -0.149856 | -2.176343 | 0.000000 |
| F | -2.775543 | -2.370885 | 0.000000 |
| H | -3.890921 | 0.002776 | 0.000000 |
| F | -2.776379 | 2.381958 | 0.000000 |
| F | -0.021390 | 2.402662 | 0.000000 |

**2,3,5-$C_6F_3H_2^{\bullet}$ (o-m-p')**
***E*** = -1549.73
***H*** = -1506.78
$N_{imag}$ = 0
**2S + 1** = 2

| | | | |
|---|---|---|---|
| C | -2.052704 | 1.209871 | 0.000000 |
| C | -0.651649 | 1.225065 | 0.000000 |
| C | -0.024704 | 0.001115 | 0.000000 |
| C | -0.671287 | -1.212251 | 0.000000 |
| C | -2.070560 | -1.226997 | 0.000000 |
| C | -2.744965 | -0.007198 | 0.000000 |
| H | -0.120373 | 2.174234 | 0.000000 |
| F | 0.012396 | -2.391666 | 0.000000 |
| H | -2.622482 | -2.163595 | 0.000000 |
| F | -4.104255 | 0.007214 | 0.000000 |
| F | -2.751122 | 2.376192 | 0.000000 |

**2,4,6-$C_6F_3H_2^{\bullet}$ (2o-p)**
***E*** = -1551.31
***H*** = -1508.32
$N_{imag}$ = 0
**2S+1** = 2

| | | | |
|---|---|---|---|
| C | -0.704533 | 1.207798 | 0.000000 |
| C | -0.046381 | -0.000000 | 0.000000 |
| C | -0.704533 | -1.207798 | 0.000000 |
| C | -2.104029 | -1.230281 | 0.000000 |
| C | -2.761276 | -0.000000 | 0.000000 |
| C | -2.104029 | 1.230281 | 0.000000 |
| F | -0.016739 | 2.381805 | 0.000000 |
| F | -0.016739 | -2.381805 | 0.000000 |
| H | -2.650605 | -2.168658 | 0.000000 |
| F | -4.126538 | -0.000000 | 0.000000 |
| H | -2.650605 | 2.168658 | 0.000000 |

**3,4,5-$C_6F_3H_2^{\cdot}$ (2m-p)**
***E*** = -1548.66
***H*** = -1505.69
$N_{imag}$ = 0
**2S+1** = 2

| | | | |
|---|---|---|---|
| C | -0.686462 | 1.233694 | 0.000000 |
| C | -0.076491 | 0.000000 | 0.000000 |
| C | -0.686462 | -1.233694 | 0.000000 |
| C | -2.087114 | -1.209227 | 0.000000 |
| C | -2.792170 | 0.000000 | 0.000000 |
| C | -2.087114 | 1.209227 | 0.000000 |
| H | -0.156292 | -2.182522 | 0.000000 |
| F | -2.794331 | -2.369750 | 0.000000 |
| F | -4.146146 | 0.000000 | 0.000000 |
| F | -2.794331 | 2.369750 | 0.000000 |
| H | -0.156292 | 2.182522 | 0.000000 |

**2,3,4,5-$C_6F_4H^{\cdot}$ (o-2m-p)**
***E*** = -1546.22
***H*** = -1507.60
$N_{imag}$ = 0
**2S + 1** = 2

| | | | |
|---|---|---|---|
| C | -0.696275 | 1.222661 | 0.000000 |
| C | -0.072296 | 0.000725 | 0.000000 |
| C | -0.688736 | -1.227259 | 0.000000 |
| C | -2.089395 | -1.209055 | 0.000000 |
| C | -2.793222 | 0.000871 | 0.000000 |
| C | -2.100628 | 1.218429 | 0.000000 |
| H | -0.154731 | -2.174307 | 0.000000 |
| F | -2.791959 | -2.371302 | 0.000000 |
| F | -4.145042 | 0.004313 | 0.000000 |
| F | -2.792404 | 2.379847 | 0.000000 |
| F | -0.026432 | 2.402265 | 0.000000 |

**2,3,5,6-$C_6F_4H^{\cdot}$ (2o-2m)**
***E*** = -1547.48
***H*** = -1508.83
$N_{imag}$ = 0
**2S+1** = 2

| | | | |
|---|---|---|---|
| C | -0.690326 | 1.220452 | 0.000000 |
| C | -0.060869 | 0.000000 | 0.000000 |
| C | -0.690326 | -1.220452 | 0.000000 |

| | | | |
|---|---|---|---|
| C | -2.095363 | -1.202694 | 0.000000 |
| C | -2.799473 | -0.000000 | 0.000000 |
| C | -2.095363 | 1.202694 | 0.000000 |
| F | -0.014276 | 2.394982 | 0.000000 |
| F | -0.014276 | -2.394982 | 0.000000 |
| F | -2.770509 | -2.382096 | 0.000000 |
| H | -3.884714 | -0.000000 | 0.000000 |
| F | -2.770509 | 2.382096 | 0.000000 |

**2,3,4,6-$C_6F_4H^{\bullet}$ (2o-m-p)**
***E*** = -1547.28
***H*** = -1508.65
$\boldsymbol{N_{imag}}$ = 0
**2S + 1** = 2

| | | | |
|---|---|---|---|
| C | -0.705513 | 1.206011 | 0.000000 |
| C | -0.054393 | -0.004414 | 0.000000 |
| C | -0.695008 | -1.219800 | 0.000000 |
| C | -2.099944 | -1.222453 | 0.000000 |
| C | -2.777712 | 0.002834 | 0.000000 |
| C | -2.104468 | 1.223432 | 0.000000 |
| F | -0.024183 | -2.395738 | 0.000000 |
| F | -2.783807 | -2.389303 | 0.000000 |
| F | -4.134786 | -0.013648 | 0.000000 |
| H | -2.653698 | 2.161059 | 0.000000 |
| F | -0.017607 | 2.379594 | 0.000000 |

**$C_6F_5^{\bullet}$ (2o-2m-p)**
***E*** = -1542.68
***H*** = -1508.37
$\boldsymbol{N_{imag}}$ = 0
**2S+1** = 2

| | | | |
|---|---|---|---|
| C | -0.693078 | 1.219396 | 0.000000 |
| C | -0.062661 | -0.000000 | 0.000000 |
| C | -0.693078 | -1.219396 | 0.000000 |
| C | -2.097271 | -1.218447 | 0.000000 |
| C | -2.787785 | -0.000000 | 0.000000 |
| C | -2.097271 | 1.218447 | 0.000000 |
| F | -0.019080 | 2.393408 | 0.000000 |
| F | -0.019080 | -2.393408 | 0.000000 |
| F | -2.785519 | -2.381454 | 0.000000 |
| F | -4.137652 | -0.000000 | 0.000000 |
| F | -2.785519 | 2.381454 | 0.000000 |

**$C_6ClH_4^{\bullet}$ (o-Cl)**
***E*** = -1504.33
***H*** = -1452.91
$\boldsymbol{N_{imag}}$ = 0
**2S+1** = 2

| | | | |
|---|---|---|---|
| C | -0.730164 | 1.204374 | 0.000000 |
| C | -2.139648 | 1.159802 | 0.000000 |
| C | -2.798945 | -0.075210 | 0.000000 |
| C | -2.082525 | -1.277660 | 0.000000 |
| C | -0.678135 | -1.236031 | 0.000000 |
| C | -0.073514 | -0.003716 | 0.000000 |

| | | | |
|---|---|---|---|
| H | -2.706457 | 2.088447 | 0.000000 |
| H | -3.885775 | -0.107366 | 0.000000 |
| H | -2.591525 | -2.238719 | 0.000000 |
| H | -0.199874 | 2.154607 | 0.000000 |
| Cl | 0.266570 | -2.728627 | 0.000000 |

**$C_6BrH_4^•$ (o-Br)**
***E*** = -1491.73
***H*** = -1440.52
$\boldsymbol{N_{imag}}$ = 0
**2S+1** = 2

| | | | |
|---|---|---|---|
| C | -0.728178 | 1.203447 | 0.000000 |
| C | -2.137616 | 1.158376 | 0.000000 |
| C | -2.799360 | -0.075703 | 0.000000 |
| C | -2.085796 | -1.280824 | 0.000000 |
| C | -0.682617 | -1.233205 | 0.000000 |
| C | -0.075881 | -0.008224 | 0.000000 |
| H | -2.704049 | 2.087331 | 0.000000 |
| H | -3.886327 | -0.105708 | 0.000000 |
| H | -2.598283 | -2.239692 | 0.000000 |
| H | -0.197002 | 2.153049 | 0.000000 |
| Br | 0.363876 | -2.863935 | 0.000000 |

**$C_6IH_4^•$ (o-I)**
***E*** = -1479.82
***H*** = -1428.75
$\boldsymbol{N_{imag}}$ = 0
**2S+1** = 2

| | | | |
|---|---|---|---|
| C | -0.727347 | 1.202258 | 0.000000 |
| C | -2.136535 | 1.158482 | 0.000000 |
| C | -2.801017 | -0.074538 | 0.000000 |
| C | -2.090652 | -1.281844 | 0.000000 |
| C | -0.685208 | -1.235624 | 0.000000 |
| C | -0.080319 | -0.013442 | 0.000000 |
| H | -2.702074 | 2.088152 | 0.000000 |
| H | -3.888230 | -0.101859 | 0.000000 |
| H | -2.609826 | -2.237130 | 0.000000 |
| H | -0.194187 | 2.150661 | 0.000000 |
| I | 0.488583 | -3.030780 | 0.000000 |

**$C_6LiH_4^•$ (o-Li)**
***E*** = -1484.86
***H*** = -1433.94
$\boldsymbol{N_{imag}}$ = 0
**2S+1** = 2

| | | | |
|---|---|---|---|
| C | -0.649668 | 0.956262 | 0.000000 |
| C | -2.049350 | 1.066943 | 0.000000 |
| C | -2.868818 | -0.077598 | 0.000000 |
| C | -2.312955 | -1.366947 | 0.000000 |
| C | -0.909725 | -1.436929 | 0.000000 |
| C | -0.131009 | -0.349503 | 0.000000 |
| H | -2.509945 | 2.054689 | 0.000000 |
| H | -3.952314 | 0.040171 | 0.000000 |
| H | -2.957673 | -2.245259 | 0.000000 |
| H | -0.026024 | 1.849677 | 0.000000 |

| | | | |
|---|---|---|---|
| Li | 1.018988 | -1.995100 | 0.000000 |

**$C_6H_4^{••}$ (o)**
***E*** = -1370.15
**2S+1** = 3

| | | | |
|---|---|---|---|
| C | -0.6999215 | 1.2122656 | 0.0000000 |
| C | 0.0000193 | -0.0000000 | 0.0000000 |
| C | -0.6999215 | -1.2122656 | 0.0000000 |
| C | -2.0997443 | -1.2122656 | 0.0000000 |
| C | -2.7996851 | -0.0000000 | 0.0000000 |
| C | -2.0997443 | 1.2122656 | 0.0000000 |
| H | -2.6437248 | -2.1545211 | 0.0000000 |
| H | -3.8876897 | -0.0000000 | 0.0000000 |
| H | -2.6437248 | 2.1545211 | 0.0000000 |
| H | -0.1559410 | 2.1545212 | 0.0000000 |

**$C_6H_4^{••}$ (m)**
***E*** = -1372.57
**2S+1** = 3

| | | | |
|---|---|---|---|
| C | -0.6999215 | 1.2122656 | 0.0000000 |
| C | 0.0000194 | -0.0000000 | 0.0000000 |
| C | -0.6999215 | -1.2122656 | 0.0000000 |
| C | -2.0997443 | -1.2122656 | 0.0000000 |
| C | -2.7996852 | -0.0000000 | 0.0000000 |
| C | -2.0997443 | 1.2122656 | 0.0000000 |
| H | -0.1559410 | -2.1545211 | 0.0000000 |
| H | -3.8876897 | -0.0000000 | 0.0000000 |
| H | -2.6437248 | 2.1545211 | 0.0000000 |
| H | -0.1559410 | 2.1545211 | 0.0000000 |

**$C_6H_4^{••}$ (p)**
***E*** = -1372.21
**2S+1** = 3

| | | | |
|---|---|---|---|
| C | -0.6999215 | 1.2122656 | 0.0000000 |
| C | 0.0000194 | -0.0000000 | 0.0000000 |
| C | -0.6999215 | -1.2122656 | 0.0000000 |
| C | -2.0997443 | -1.2122656 | 0.0000000 |
| C | -2.7996852 | -0.0000000 | 0.0000000 |
| C | -2.0997443 | 1.2122656 | 0.0000000 |
| H | -0.1559410 | -2.1545211 | 0.0000000 |
| H | -2.6437248 | -2.1545211 | 0.0000000 |
| H | -2.6437248 | 2.1545211 | 0.0000000 |
| H | -0.1559410 | 2.1545211 | 0.0000000 |
